# The Association of *HLA-G* Gene Polymorphism and Its Soluble Form With Male Infertility

**DOI:** 10.3389/fimmu.2021.791399

**Published:** 2022-01-17

**Authors:** Karolina Piekarska, Paweł Radwan, Agnieszka Tarnowska, Andrzej Wiśniewski, Rafał Krasiński, Michał Radwan, Jacek R. Wilczyński, Andrzej Malinowski, Izabela Nowak

**Affiliations:** ^1^ Department of Clinical Immunology, Laboratory of Immunogenetics and Tissue Immunology, Ludwik Hirszfeld Institute of Immunology and Experimental Therapy, Polish Academy of Sciences, Wrocław, Poland; ^2^ Department of Reproductive Medicine, Gameta Hospital, Rzgów, Poland; ^3^ Faculty of Health Sciences, The State University of Applied Sciences in Płock, Płock, Poland; ^4^ Department of Surgical and Oncological Gynecology, Medical University of Łódź, Łódź, Poland; ^5^ Department of Surgical, Endoscopic and Oncologic Gynecology, Polish Mothers’ Memorial Hospital–Research Institute, Łódź, Poland

**Keywords:** HLA-G polymorphism, sHLA-G, male infertility, *In vitro* fertilization, semen

## Abstract

Successful reproduction depends on many factors. Male factors contribute to infertility in approximately 50% of couples who fail to conceive. Seminal plasma consists of secretions from different accessory glands containing a mixture of various cytokines, chemokines, and growth factors, which together can induce a local immune response that might impact on a male’s as well as a female’s fertility. Human leukocyte antigen (HLA)-G expression has been suggested as an immunomodulatory molecule that influences pregnancy outcome. The *HLA-G* gene encodes either membrane-bound or/and soluble proteins. The aim of this study was the evaluation of HLA-G polymorphisms and their impact on soluble HLA-G (sHLA-G) production. We tested the *HLA-G* polymorphism in three positions: rs1632947: c.-964G>A; rs1233334: c.-725G>C/T in the promoter region; rs371194629: c.∗65_∗66insATTTGTTCATGCCT in the 3′ untranslated region. We tested two cohorts of men: 663 who participated in *in vitro* fertilization (test material was blood or sperm), and 320 fertile controls who possessed children born after natural conception (test material was blood). Since 50% of men visiting assisted reproductive clinics have abnormal semen parameters, we wondered if men with normal sperm parameters differ from those with abnormal parameters in terms of *HLA-G* polymorphism and secretion of sHLA-G into semen. We found that certain rs1632947-rs1233334-rs371194629 *HLA-G* haplotypes and diplotypes were associated with male infertility, while others were protective. Normozoospermic men with the A-C-del haplotype and A-C-del/A-C-del diplotype secreted the most sHLA-G into semen (574.1 IU/mL and 1047.0 IU/mL, respectively), while those with the G-C-ins haplotype and G-C-ins/G-C-ins diplotype – the least (80.8 IU/mL and 75.7 IU/mL, respectively). Men with the remaining haplotypes/diplotypes secreted sHLA-G at an intermediate level. However, only in one haplotype, namely G-C-ins, did we observe strong significant differences in the concentration of sHLA-G in the semen of men with teratozoospermia compared to men with normal sperm parameters (p = 0.009). In conclusion, fertile men differ in the profile of *HLA-G* polymorphism from men participating in IVF. Among all *HLA-G* haplotypes, the most unfavorable for male fertility is the G-C-ins haplotype, which determines the secretion of the lowest concentration of the soluble HLA-G molecule. This haplotype may reduce sperm parameters.

## Introduction

Successful reproduction depends on many factors. Male factors contribute to infertility in approximately 50% of couples who fail to conceive (1). This is a growing problem observed around the world and in Poland (2). Decreased semen quality has been observed over the years and may be caused by endocrine disrupting chemicals (3, 4) but it can also result from anatomical or genetic abnormalities, systemic or neurological diseases, infections, trauma, iatrogenic injury, gonadotoxins and the development of sperm antibodies. Male infertility may be due to testicular and post-testicular deficiencies. However, in 30–40% of male infertility cases, no cause is identified (idiopathic male infertility) (5). Moreover, male factors may have an influence upon fertilization and embryo development failure, the increase in the risk of idiopathic recurrent miscarriages, autosomal dominant diseases and neurobehavioral disorders in their offspring (6).

Seminal plasma consists of secretions from different accessory glands, such as the epididymis, seminal vesicle, prostate, and bulbourethral gland. Seminal plasma contains a heterogeneous mixture of various cytokines, chemokines, and growth factors, which together can induce a local immune response that might impact on a male’s as well as a female’s fertility. These molecules are considered crucial for spermatocyte transport, function and protection against sperm damage (7–11).

So far, only a few studies have revealed that seminal fluid contains varying amounts of soluble HLA‐G (sHLA‐G) protein (12–16). The level of sHLA-G depends on the *HLA-G* genotype and haplotype (16, 17). The *HLA-G* gene encodes either membrane-bound and/or soluble proteins due to alternative splicing of its transcript: HLA-G1 to HLA-G4 are membrane bound, while HLA-G5 to HLA-G7 soluble. The most important isoforms are the full-length membrane bound HLA-G1 and the full-length secreted HLA-G5. Soluble HLA-G1 (sHLA-G1) is generated by the shedding of membrane-bound HLA-G1 molecules (18).

*HLA-G* gene, in contrast to classical HLA class I gene, is highly polymorphic in the non-coding 3′ untranslated region (UTR) and in the 5′ upstream regulatory region (5′URR) (19, 20). The 14 bp insertion/deletion polymorphism (rs371194629 c. ∗ 65_ ∗ 66insATTTGTTCATGCCT) is between positions +2961 and +2974. A 14 bp insertion is considered an ancestral allele and a small fraction of the transcripts have been reported to undergo further alternative splicing leading to the removal of 92 bases (including 14 bases) from the 5’ end of the previously named “exon 8”. The 92 bases deleted transcripts are more stable than the longer ones (21). The insertion allele is associated with lower levels or absence of sHLA-G in plasma (19, 22–24). HLA-G expression is determined by the combination of multiple single nucleotide polymorphisms (SNPs) (24, 25). Therefore, in addition to the 14-bp insertion/deletion in 3′UTR, we tested other polymorphic positions in the *HLA-G* gene promoter region, which could affect the level of HLA-G expression, rs1632947: c.-964G>A and rs1233334: c.-725G>C/T, and to correlate them with the level of soluble HLA-G secreted into the plasma of semen. The presence of an adenine at -964 position (CpA dinucleotide) destroys a potentially methylated CpG dinucleotide (26), and the G variant at position -725 (C>G, T) creates a CpG dinucleotide influencing the transcriptional activity of the gene (27). However, Ober et al. revealed a significantly higher expression level of the promoter haplotype containing the -725G allele compared with those containing the -725C or -725T alleles (28).

The aim of this study was the evaluation of the role of sHLA-G and its gene polymorphism in male infertility. Since 50% of men visiting assisted reproductive clinics have abnormal semen parameters, we were curious if men with normal sperm parameters differed from those with abnormal parameters in terms of secreting sHLA-G into semen.

## Material and Methods

### Study Design

In our research, we tested a total of 993 men. Six hundred and sixty three males were patients who, together with their female partners, underwent *in vitro* fertilization (IVF), while a group of 320 fertile men constituted the control group. From the IVF group, we collected blood from 480 men, and semen from 183 men. Patients were qualified at the Gameta Assisted Reproduction Clinic in Rzgów, certified by the European Society for Human Reproduction and Embryology (ESHRE). Patients were also recruited from the Department of Surgical, Endoscopic and Oncologic Gynecology and the Department of Gynecology and Gynecologic Oncology, Polish Mothers’ Memorial Hospital–Research Institute in Łódź and Gynemed. The men were of mean age 35.06 years ± 5.08 (age range 19-53). The semen samples were obtained by masturbation after 2-7 days of sexual abstinence. The patients’ ejaculate samples were analyzed and categorized according to the nomenclature of the WHO (World Health Organization) from 2010 using Sperm Class Analyzer CASA System (29). Normozoospermia means the total number of sperm cells, their concentration, progressively motile and morphology above or equal reference values (N ≥ 15 mln/mL of sperm cells); OS – oligozoospermia; Moderate OS (5 < N < 15 mln/mL); Severe OS (1-5 mln/mL), very severe OS (N < 1 mln/mL); AS – azoospermia (lack of sperm cells in ejaculate); Asthenozoospermia – number of sperm cells with progressive motility below reference values; Teratozoospermia – number of morphologically normal sperm cells below reference values.

The control group was recruited mainly from the 1st Department of Obstetrics and Gynecology, Medical University of Warsaw. These men and their female partners had at least one healthy child from natural conception. The men were of mean age 34.15 years ± 6.29 (age range 25-70). All tested males were of Polish origin. Men from the control group differ in age from IVF men (p = 0.0003).

Experimental protocols were approved by Local Ethics Committees (the agreement of Medical University of Wrocław and Polish Mothers’ Memorial Hospital–Research Institute in Łódź) and informed consent was obtained from all individual participants included in the study.

### DNA Preparation and Genotyping

Genomic DNA was isolated from venous blood and semen using the Invisorb Spin Blood Midi Kit (Invitek, Germany) or QIAamp DNA Blood Mini Kit (Qiagen, Germany) or NucleoSpin Blood, Mini kit for DNA from blood (Macherey-Nagel, Germany) according to the manufacturer’s instructions. We tested HLA-G polymorphism in three positions: rs1632947: c.-964G>A, rs1233334: c.-725G>C/T in the promoter, and rs371194629: c.∗65_∗66insATTTGTTCATGCCT in 3′UTR. Polymorphisms in the *HLA-G* gene were performed in all qualified men by PCR-SSP method or using TaqMan assays in Real-Time PCR according to Bylinska et al. and Nowak et al. (17, 30).

### sHLA-G Measurement

We had access to 183 semen samples taken from patients. Samples were stored at −80°C until the time of assay. The concentration of sHLA-G (IU/mL) in semen plasma was tested with a sandwich enzyme-linked immunosorbent assay (ELISA) kit following the manufacturer’s protocol (Exbio/Biovendor, Czech Republic). Standard curve measured the concentration of HLA-G1 (which are shed from the cell surface by proteolytic cleavage) and sHLA-G5 isoforms from 3.91 to 125 IU/mL. The limit of sHLA-G detection in this test was 0.6 IU/mL. Samples in which the sHLA-G concentration exceeded 125 IU/mL were retested after diluting them by a factor 1:4.

### Statistical Analysis

We used the two-tailed Fisher exact test (R program version 3.4.3) for the estimation of allelic and genotypic frequencies. Deviation of the genotype counts from Hardy-Weinberg equilibrium was analyzed using the chi-square test with one degree of freedom. All genotype frequencies were in Hardy-Weinberg equilibrium both in control and in patient groups. A p < 0.05 was considered significant. The odds ratio (OR) and its 95% CI were computed as the measure of effect size. For multiple comparison tests, the Bonferroni correction was done. Haplotypes were generated by FAMHAP 19 (http://famhap.meb.uni-bonn.de). Statistical analyses concerning sHLA-G concentration measured in the seminal plasma of patients were performed using Mann-Whitney or T-student test (GraphPad Prism 5 software). All parameters of statistical analyses concerning sHLA-G (numbers, medians, means, standard deviation and errors, min, max, and 25-75% percentiles) are part of **Supplementary Tables 6** **–** **8**. The power calculation for the Mann-Whitney test was estimated using the available free calculator https://www.benchmarksixsigma.com/calculators/sample-size-calculator-for-mann-whitney-test/. This calculator allowed us to determine the number of samples needed to compare two population medians with at least 80% test power and a confidence level of 95%.

## Results

### *HLA-G* Polymorphism in Fertile Men and Men Participating in IVF

We did not find differences in the frequencies of the individual *HLA-G* genotypes (**Supplementary Table 1**). Instead, we observed differences in the frequencies of haplotypes. Among controls and patients, 9 haplotypes in the following order rs1632947–rs1233334–rs371194629 were generated. Out of 9 detected haplotypes, 2 of them (A-C-ins and G-G-del) were statistically significantly more frequent in the group of fertile men compared to men participating in *in vitro* fertilization (p < 0.0001/p_corr._ = 0.0004, OR = 0.653, 95% CI = 0.53-0.80; p = 0.005/p_corr._ = 0.042, OR = 0.648, 95% CI = 0.48-0.88, respectively, **Table 1**). Two haplotypes (A-G-del and G-C-ins) were also observed to be carried more frequently by men from the IVF group than men from the control group (p < 0.0001/p_corr._ < 0.0001, OR = 3.535, 95% CI = 1.73-8.14; p < 0.0001/p_corr._ < 0.0001, OR = 3.035, 95% CI = 2.11-4.47, respectively, **Table 1**)

**Table 1:** *HLA-G* haplotype frequencies in men from Control and IVF group.

Haplotype^*^	Fertile control (%)	IVF men (%)	IVF men vs. Fertile control		
	2N = 638	2N = 1308	p/p_corr._	OR	95% CI
ACdel	90 (14.11)	216 (16.51)	0.185	1.204	0.92-1.59
ACins	229 (35.89)	350 (26.76)	**<0.0001/0.0004**	0.653	0.53-0.80
AGdel	9 (1.41)	63 (4.82)	**<0.0001/0.0001**	3.535	1.73-8.14
ATdel	1 (0.16)	9 (0.69)	**0.181**	4.412	0.61-193.59
GCdel	170 (26.65)	315 (24.08)	**0.220**	0.873	0.70-1.09
GCins	38 (5.96)	211 (16.13)	**<0.0001/<0.0001**	3.035	2.11-4.47
GGdel	86 (13.48)	120 (9.17)	**0.005/0.042**	0.648	0.48-0.88
GGins	0 (0.00)	3 (0.23)	0.555	–	–
GTins	15 (2.35)	21 (1.61)	0.283	0.678	0.33-1.42

^*^Haplotypes were estimated in the following order: rs1632947:-964G>A; rs1233334:-725G>C/T; rs371194629:insATTTGTTCATGCCT/del. Values in bold indicate signiﬁcant differences. IVF, in vitro fertilization; p, probability; p_corr._, probability after Bonferroni correction for 9 possible haplotypes; OR, odds ratio; 95% CI, confidence interval from two-sided Fisher’s exact test; ns, not significant.

We also estimated 23 different diplotypes: 19 of them were present in the control group, while 21 were present in the IVF group. To determine the diplotypes, we adopted the same order of tested SNPs as in the case of haplotypes (**Table 2**). For 4 diplotypes, we found differences in frequencies between IVF and controls. Diplotypes A-C-del/A-G-del and G-C-ins/G-C-ins were more prevalent in patients than controls (p = 0.0001/p_corr._ = 0.002, OR = 4.857, 95% CI = 1.91-15.81; p < 0.0001/p_corr._ = 0.001, OR = 3.066, 95% CI = 1.72-5.82, respectively, **Table 2**). Two diplotypes (A-C-ins/A-C-ins and G-G-del/G-C-del) were protective and present in higher frequencies in the control group than in comparison to the IVF group (p < 0.0001/p_corr._ < 0.0001, OR = 0.273, 95% CI = 0.16-0.46; p < 0.0001/p_corr._ < 0.0001, OR = 0.191, 95% CI = 0.08-0.42, respectively, **Table 2**). Additionally, we found a significant association for the G-C-del/G-C-ins (p = 0.024/p_corr._ = ns, OR = 2.396, 95% CI = 1.08-6.02, **Table 2**). A protective effect was observed for A-C-ins/A-C-del (p = 0.039/p_corr._ = ns, OR = 0.474, 95% CI = 0.22-1.00) and G-T-ins/A-C-del (p = 0.035/p_corr._ = ns, OR = 0.000, 95% CI = 0.00-1.18, **Table 2**). However, statistical significance in these analyses was lost when the correction for multiple comparisons was applied.

**Table 2:** *HLA-G* diplotype frequencies in men from Control and IVF groups.

Diplotype^*^	Fertile control (%)	IVF men (%)	IVF men vs. Fertile control		
	N = 319	N = 654	p/p_corr._	OR	95% CI
AGdel/AGdel	2 (0.63)	8 (1.22)	0.512	1.962	0.39-19.07
ACdel/AGdel	5 (1.57)	47 (7.19)	**0.0001/0.002**	4.857	1.91-15.81
ACdel/ACdel	15 (4.70)	45 (6.88)	0.204	1.497	0.80-2.94
ACins/ACdel	17 (5.33)	17 (2.60)	**0.039/ns**	0.474	0.22-1.00
ACins/ACins	45 (14.11)	28 (4.28)	**<0.0001/<0.0001**	0.273	0.16-0.46
GGdel/GGdel	5 (1.57)	3 (0.46)	0.123	0.290	0.04-1.50
GGdel/GCdel	24 (7.52)	10 (1.53)	**<0.0001/0.0001**	0.191	0.08-0.42
GCdel/GCdel	20 (6.27)	28 (4.28)	0.207	0.669	0.36-1.27
GCdel/GCins	8 (2.51)	38 (5.81)	**0.024/ns**	2.396	1.08-6.02
GCins/GCins	15 (4.70)	86 (13.15)	**<0.0001/0.001**	3.066	1.72-5.82
ACdel/GCdel	22 (6.90)	45 (6.88)	1.000	0.998	0.57-1.78
ACdel/GGdel	13 (4.08)	16 (2.45)	0.165	0.591	0.26-1.35
ACins/ATdel	1 (0.31)	8 (1.22)	0.285	3.934	0.52-175.16
ACins/GCdel	75 (23.51)	164 (25.08)	0.634	1.089	0.79-1.51
ATdel/ACdel	0 (0.00)	1 (0.15)	1.000	–	–
GCins/ACins	0 (0.00)	1 (0.15)	1.000	–	–
GGdel/ACins	37 (11.60)	87 (13.30)	0.475	1.169	0.76-1.82
GGdel/GGins	0 (0.00)	1 (0.15)	1.000	–	–
GGins/GTins	0 (0.00)	2 (0.31)	1.000	–	–
GTins/ACdel	3 (0.94)	0 (0.00)	**0.035/ns**	0.000	0.00-1.18
GTins/ACins	9 (2.82)	17 (2.60)	0.834	0.919	0.38-2.37
GTins/GCdel	1 (0.31)	2 (0.31)	1.000	0.975	0.05-57.71
GTins/GGdel	2 (0.63)	0 (0.00)	0.107	0.000	0.00-2.59

*Diplotypes were estimated in the following order: rs1632947:-964G>A; rs1233334:-725G>C/T; rs371194629:insATTTGTTCATGCCT/del. Values in bold indicate signiﬁcant differences. IVF, in vitro fertilization; p, probability; p_corr._, probability after Bonferroni correction for 23 possible diplotypes; OR, odds ratio; 95% CI, confidence interval from two-sided Fisher’s exact test; ns, not significant.

Chi-square for trend: ACdel/ACdel vs. ACins/ACdel vs. ACins/ACins: p < 0.0001; GCdel/GCdel vs. GCdel/GCins vs. GCins/GCins p = 0.0005.

When we compared diplotypes which differed only in the insertion allele (G-C-del/G-C-del vs. G-C-del/G-C-ins vs. G-C-ins/G-C-ins), we observed a strong association with male infertility expressed by a higher frequency in samples from the IVF group (p = 0.0005; Chi-square test for trend). The insertion allele in these diplotypes was disadvantageous because the odds ratios increased from protective 0.669 in G-C-del/G-C-del men to predisposing 2.396 in GC-del/G-C-ins men and 3.066 in G-C-ins/G-C-ins men. However, the insertion allele in diplotypes A-C-ins/A-C-del and A-C-ins/A-C-ins was protective, and the odds ratios ranged from 1.497 in A-C-del/A-C-del patients to 0.474 in A-C-ins/A-C-del and to 0.273 in A-C-ins/A-C-ins patients (p < 0.0001, Chi-square test for trend; **Table 2**).

We can conclude that fertile men differ in the profile of *HLA-G* polymorphism from men participating in IVF.

### *HLA-G* Polymorphism and Stratification of Patients According to Sperm Parameters

When patients were stratified by sperm count, i.e., normozoospermia, moderate, severe and very severe oligozoospermia, we did not observe differences in haplotype frequencies with the exception of the G-T-ins haplotype, which was more common in the groups with a low sperm count and no sperm (p = 0.013/p_corr._ = ns, OR = 3.140, 95% CI = 1.18-9.26, p = 0.006/p_corr._ = ns, OR = 4.068, 95% CI = 1.33-13.01 respectively, **Supplementary Table 2**). By dividing the patients into those with all normal parameters and those with abnormal parameters, we found differences in the frequency of G-C-ins haplotype (p = 0.009/p_corr._ = ns, OR = 1.525, 95% CI = 1.10-2.12, **Supplementary Table 3**). In particular, a stronger association for this haplotype was observed when comparing the male with teratozoospermia and normozoospermia. (p = 0.0005/p_corr._ = 0.004, OR = 2.000, 95% CI = 1.34-2.99, **Supplementary Table 3**).

After analysis of diplotype frequencies, only G-T-ins/A-C-ins was more frequent in the group with a reduced sperm count and no sperm than in men with normal number of sperm cells (p = 0.011/p_corr._ = ns, OR = 3.829, 95% CI = 1.24-14.06, p = 0.012/p_corr._ = ns, OR = 4.500, 95% CI = 1.20-18.35, respectively, **Supplementary Table 4**). Some associations were found in diplotype analysis of patients with reduced sperm motility or morphology (**Supplementary Table 5**). Namely, the A-C-ins/A-C-ins and G-C-del/G-C-del diplotypes were more common in men with normal sperm parameters than in men with teratozoospermia (p = 0.026/p_corr._ = ns, OR = 0.135, 95% CI = 0.00-0.90 and p = 0.044/p_corr._ = ns, OR = 0.157, 95% CI = 0.00-1.07, respectively). Conversely, the G-C-ins/G-C-ins diplotype was more common in men with teratozoospermia (p = 0.008/p_corr._ = ns, OR = 2.224, 95% CI = 1.19-4.15, **Supplementary Table 5**).

### Impact of HLA-G Haplotypes/Diplotypes on Soluble HLA-G Level in Semen of Patients Participating in IVF

We tested 183 semen samples for sHLA-G secretion. Regardless of the haplotype in the *HLA-G* gene, men with normozoospermia secreted more sHLA-G (median 288.9 IU/mL) than men with abnormal sperm parameters (median 227.9 IU/mL). This difference was not statistically significant (**Supplementary Table 6**).

**Table 3** shows the level of sHLA-G measured in semen depending on the presence of haplotypes and diplotypes in men with normal and abnormal semen parameters. Due to the insufficient number of samples having A-T-del and G-T-ins haplotype, the sHLA-G level in these samples cannot be interpreted. Normozoospermic men with the A-C-del haplotype secreted the most sHLA-G into semen (574.1 IU/mL), while those with the G-C-ins haplotype – the least (80.8 IU/mL). Men with the remaining haplotypes secreted sHLA-G at an intermediate level. When analyzing men with regards to diplotype, we can conclude that the carriers of the diplotype A-C-del/A-C-del secreted the most (1047.0 IU/mL), while the carriers of G-C-ins/G-C-ins – the least (75.7 IU/mL).

**Table 3:** The level of secreted HLA-G in semen dependent on the haplotype/diplotype in normozoospermic men and men with sperm abnormalities.

Haplotype/diplotype*	Normozoospermia		Abnormal sperm	
	N	median [IU/mL]	N	median [IU/mL]
ATdel	2	847.1	1	594.9
ACdel	22	574.1	47	650.0
ACins	23	391.2	50	261.7
GCdel	25	347.7	47	351.5
AGdel	12	315.2	5	395.3
GGdel	7	145.9	21	200.7
GCins	28	80.8	70	67.5
GTins	1	31.3	3	178.8
ACdel/ACdel	6	1 047.0	16	743.4
ACins/ATdel	2	847.1	1	594.9
ACins/GCdel	13	720.1	29	495.9
AGdel/AGdel	2	582.2	0	–
ACdel/GCdel	2	537.9	6	459.4
ACdel/AGdel	8	315.2	5	395.3
GCdel/GCdel	1	160.6	1	91.1
GGdel/ACins	7	145.9	17	99.8
GCdel/GCins	8	112.5	10	149.7
GCins/GCins	10	75.7	30	59.6
GTins/ACins	1	31.3	3	178.8
ACdel/GGdel	0	–	4	282.8

*Haplotypes/diplotypes were estimated in the following order: rs1632947:-964G>A; rs1233334:-725G>C/T; rs371194629:insATTTGTTCATGCCT/del. Normozoospermia – total number of sperm cells, their concentration, progressive motility and morphology above or equal reference values; Men with abnormal sperm – men with at least one parameter of semen below reference value.

**Figure 1:**
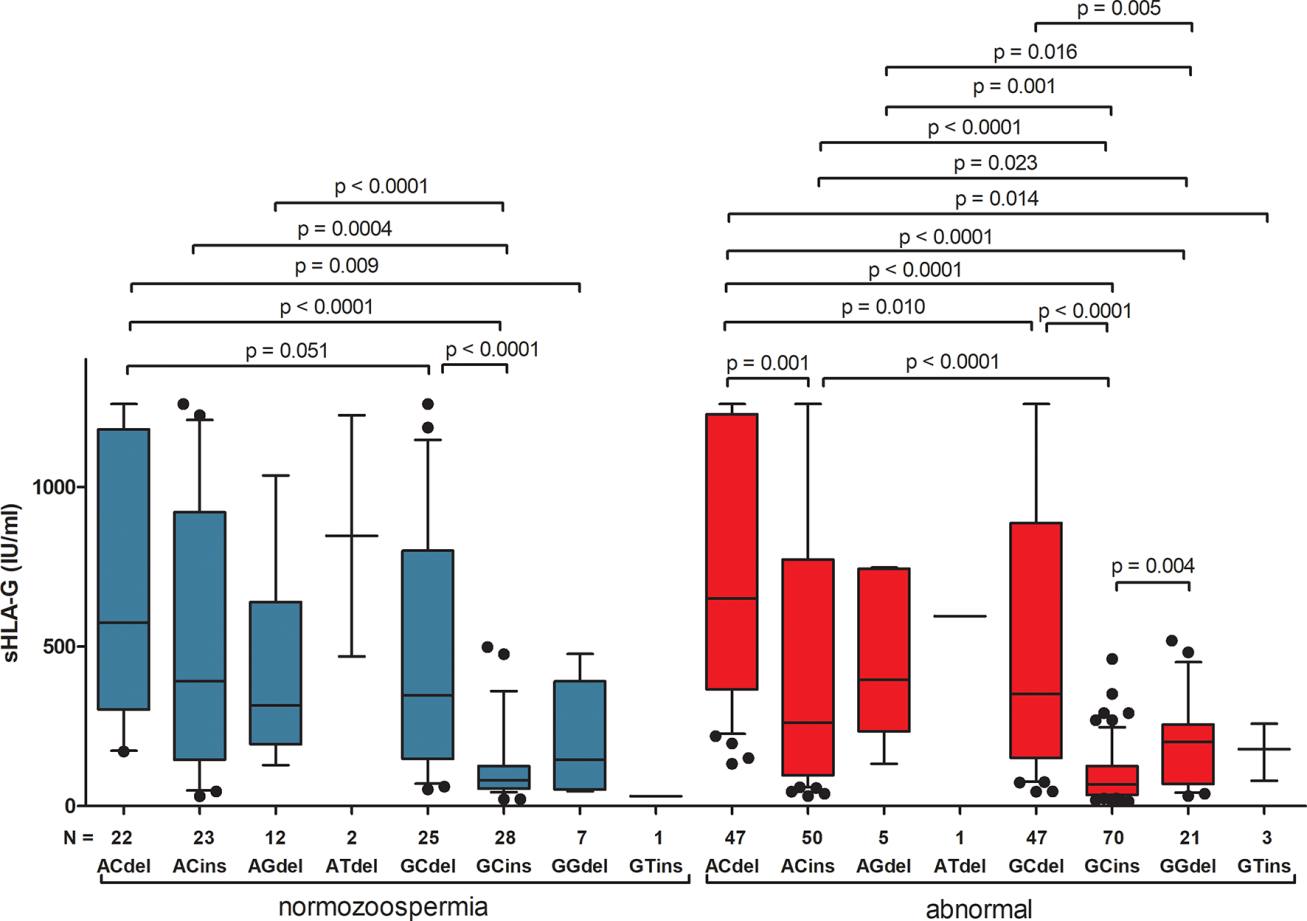
Concentration of soluble HLA-G per milliliter of plasma (IU/ml) measured in semen samples according to *HLA-G* haplotypes. Haplotypes were estimated in the following order: rs1632947:−964G>A; rs1233334:−725G>C/T; rs371194629:insATTTGTTCATGCCT/del. Blue boxes represent the level of sHLA-G measured in normozoospermic men and red boxes – in men with abnormal semen parameters. Boxes are drawn from the first quartile (25th Percentile) to the third quartile (75th Percentile). Black lines in boxes are medians. Whiskers represent 10-90 percentiles. N is the number of patients. P-values are calculated by Mann-Whitney test or unpaired t test.

**Figure 2:**
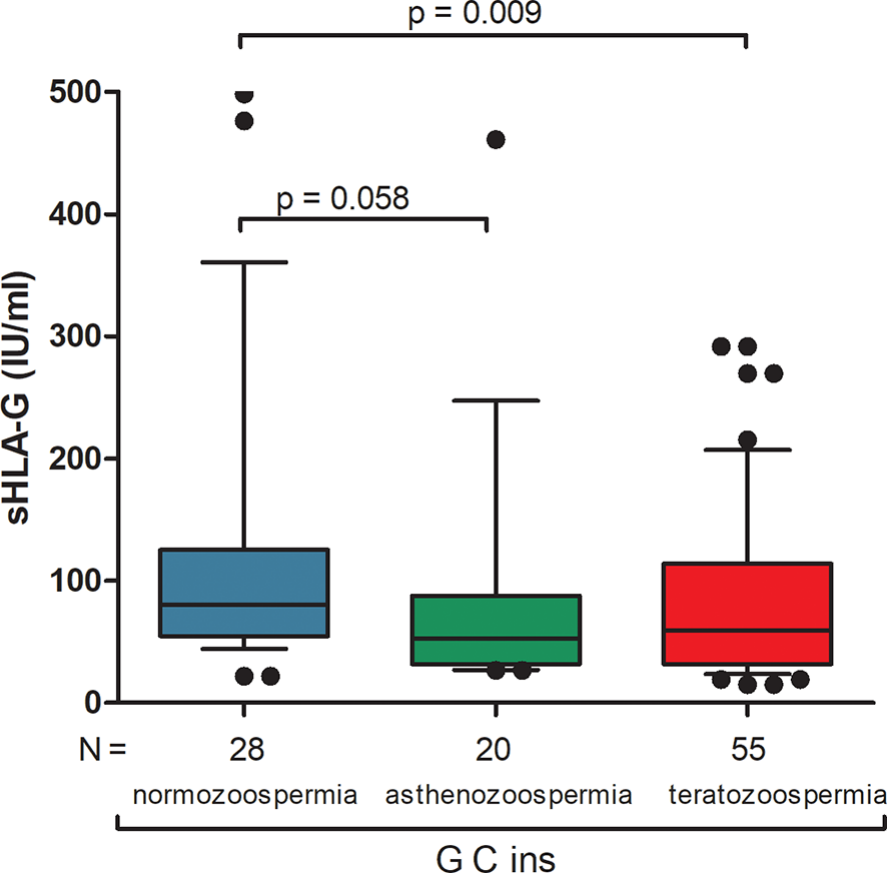
Concentration of soluble HLA-G per milliliter of plasma (IU/ml) measured in semen samples of G-C-ins haplotype carriers. Haplotype was estimated in the following order: rs1632947:−964G>A; rs1233334:−725G>C/T; rs371194629:insATTTGTTCATGCCT/del. Blue box represents the level of sHLA-G measured in normozoospermic men, green box – in men with asthenozoospermia, red box – in men with teratozoospermia. Boxes are drawn from the first quartile (25th Percentile) to the third quartile (75th Percentile). Black lines in boxes are medians. Whiskers represent 10-90 percentiles. N is the number of patients. P-values are calculated by Mann-Whitney test.

Differences in secretion of sHLA-G depending on the haplotype are also shown in **Figure 1** and **Supplementary Table 7**. The greatest differences in sHLA-G concentration were observed when comparing the G-C-ins haplotype (due to being the one that determines lowest secretion) with the remaining haplotypes in men with normozoospermia (G-C-ins vs. A-C-del p < 0.0001, G-C-ins vs. A-C-ins p = 0.0004, G-C-ins vs. A-G-del p < 0.0001; G-C-ins vs. G-C-del p < 0.0001). This regularity is also visible for men with abnormal sperm parameters. We did not observe differences in individual haplotypes between men with normozoospermia and men with abnormal sperm parameters, as well as after dividing patients into asthenozoospermic and teratozoospermic groups. Only in one haplotype, namely G-C-ins, did we observe strong significant differences in the concentration of sHLA-G in the semen of men with teratozoospermia compared to men with normal sperm parameters (p = 0.009). Men with the G-C-ins haplotype with asthenozoospermia also secreted less sHLA-G, but this difference lost significance (p = 0.058) (**Figure 2**).

**Figure 3:**
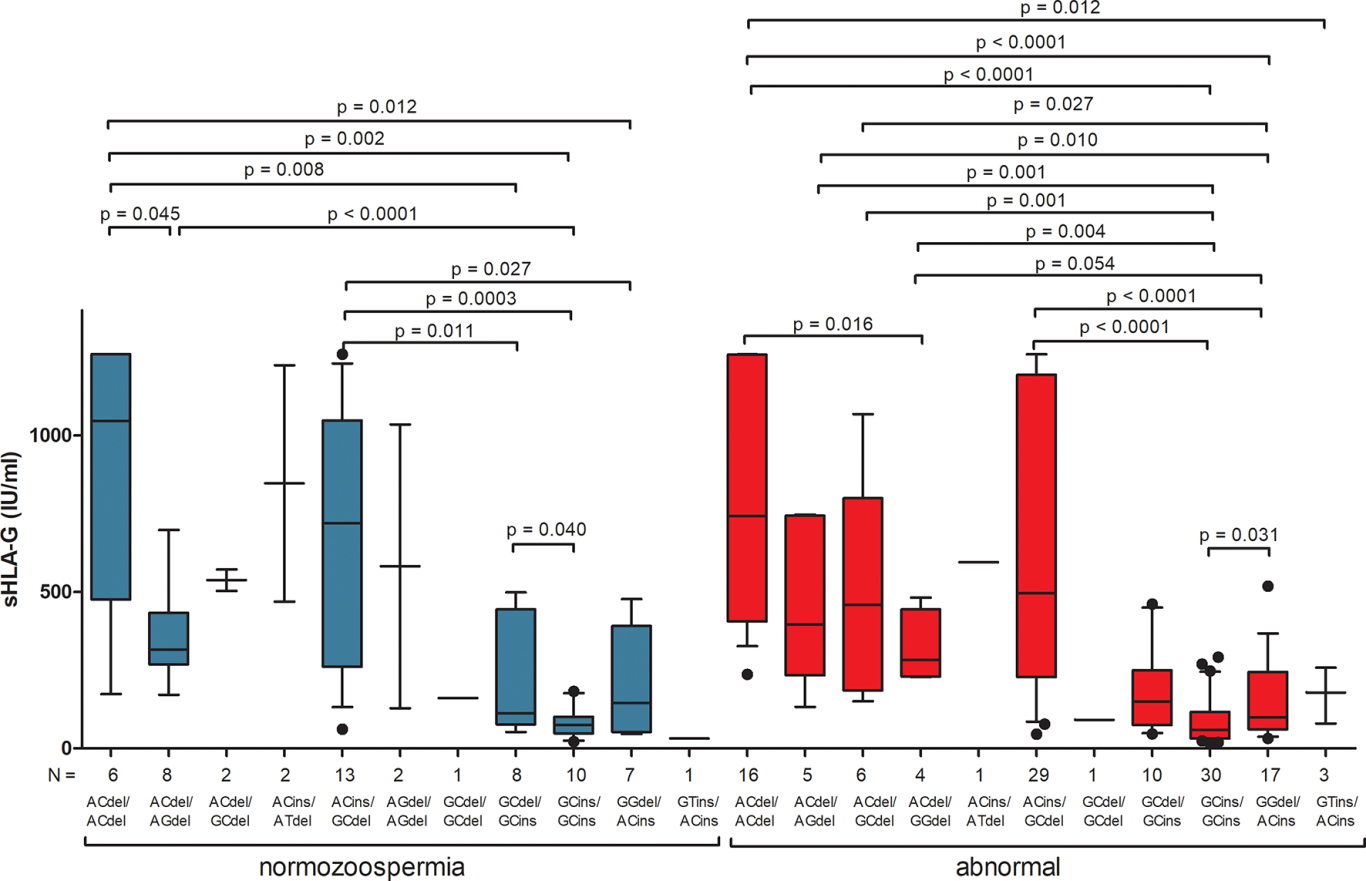
Concentration of soluble HLA-G per milliliter of plasma (IU/mL) measured in semen samples according to *HLA-G* diplotypes. Diplotypes were estimated in the following order: rs1632947:−964G>A; rs1233334:−725G>C/T; rs371194629:insATTTGTTCATGCCT/del. Blue boxes represent the level of sHLA-G measured in normozoospermic men and red boxes – in men with abnormal semen parameters. Boxes are drawn from the first quartile (25th Percentile) to the third quartile (75th Percentile). Black lines in boxes are medians. Whiskers represent 10-90 percentiles. N is the number of patients. P-values are calculated by Mann-Whitney test or unpaired T-test.

For most analyses of sHLA-G secretion depending on diplotypes, the results cannot be interpreted due to insufficient numbers of individual diplotypes, which is visible in **Figure 3** and **Supplementary Table 8**. As for haplotypes, there are significant differences in sHLA-G secretion between individual diplotypes but within one group of men, not between men with normozoospermia and men with abnormal sperm parameters. However, when we compared secretion of sHLA-G in patients with rs1632947G allele, who differed only by the insertion allele (G-C-del/G-C-del vs. G-C-del/G-C-ins vs. G-C-ins/G-C-ins), we found significant differences among these diplotypes in men with abnormal sperm parameters (p = 0.031, Kruskal-Wallis test), while in comparisons of diplotypes A-C-del/A-C-del vs. A-C-ins/A-C-del vs. A-C-ins/A-C-ins, such differences were not observed (**Supplementary Table 8**, **Figure 3**).

## Discussion

We tested two cohorts of men: those who participated in *in vitro* fertilization (test material was blood or sperm), and fertile controls who had children from natural conception (test material was blood). We found that certain rs1632947-rs1233334-rs371194629 *HLA-G* haplotypes and diplotypes were associated with male infertility, while others were protective. A-C-ins and G-G-del haplotypes were significantly more common in the fertile control group compared to men participating in *in vitro* fertilization, while the opposite trend was observed for haplotypes – A-G-del and G-C-ins. The role of G-C-ins haplotype seems to be crucial due to the fact that this haplotype determines the lowest secretion of sHLA-G and its association has been observed in men with teratozoospermia. However, the test power calculator indicated that we need 318 male samples with the G-C-ins haplotype (additionally 270 samples) to achieve a test power of 80% and a confidence level of 95% to compare the medians of men with normozoospermia and asthenozoospermia. When comparing men with normozoospermia and teratozoospermia (p = 0.009), we need 240 persons to achieve this power. Assuming a G-C-ins haplotype frequency in IVF men of 16.13% (**Table 1**), we should still have 1,928 samples to find 318 with this haplotype.

According to previous data regarding HLA-G linkage disequilibrium obtained in Brazil and other countries: A-C-del haplotype corresponds to G*01:04 allele, G0104 and UTR-3; A-C-ins haplotype – G*01:01:02, G*01:06 or G*01:05N, G010102 promoter, UTR-2; haplotype G-C-del – G*01:01:01:01 or G*01:01:01:04, UTR-1 or UTR-6; haplotype G-G-del – Promo G010101b and G010101c, G*01:01:01:04 and G*01:01:01:05, UTR-4 and UTR-6; haplotype G-T-ins, G*01:03, UTR-5, Promo G0103 (22, 24). According to Castelli et al. (24) G-C-ins haplotype corresponds to the G010101 (a, b, c, d, f), G010102a, G010104 (a, b) and UTR-2, UTR-5, UTR-7 alleles. G-G-ins and A-G-del haplotypes are very rare in general population, including G-C-ins (below 1%). By contrast, G-C-ins haplotype in our fertile male population are at the level of 5.96%. In fertile women, this percentage is 0.31% (only 2 women out of 320 controls, for details see reference 17). This points to gender differences. Our control groups (women and their partners) do not, however, reflect the randomly selected population group in which there are both fertile and infertile subjects. Our groups were specifically selected for research into fertility and human reproductive diseases. Whether we isolate DNA from blood or sperm should not affect the genotype of a given person and should not affect the results, although in our semen isolates we have more G-C-ins haplotype than would be expected compared to the frequency for the world population. Testing another semen cohort could help to overcome this issue and, as previously mentioned, would enhance the power of the test.

Our genetic and protein studies suggest the involvement of sHLA-G in seminal plasma in the creation of an immune environment suitable for maintaining the proper parameters of sperm required for optimal oocyte fertilization. There are reports showing the HLA-G molecule is important for facilitating fertilization of the oocyte and promoting successful embryo implantation (7, 12, 14). The presence of HLA-G mRNA was found in unfertilized oocytes and in early embryos. Expression of HLA-G mRNA was associated with an increased cleavage rate, when compared to embryos lacking the HLA-G transcript (31–34). If the paternal G-C-ins haplotype is inherited by the embryo, this may result in lower sHLA-G production. Our unpublished data indicate that embryos whose transfer to the uterus resulted in pregnancy secreted twice as much sHLA-G as those embryos whose transfer ended in no pregnancy or miscarriage.

Several studies have suggested an immunoregulatory role of HLA-G in the male reproductive system and in seminal plasma. Larsen et al. detected sHLA-G protein in seminal plasma, and HLA-G expression in normal testis and in epididymal tissue of the male reproductive system but not in the seminal vesicle (12). In the testis, HLA-G might have a role as an immunosuppressive factor, and thereby avoiding recognition of ‘self’ sperm cells, which can be perceived as autoantigens by the immune system. On the other hand, paternal sHLA-G in the seminal plasma, may be associated with the induction of tolerance in the mother to paternal antigens. This induction may be important for the success of the pregnancy. Costa et al. enrolled couples undergoing assisted reproduction treatment and couples who conceived naturally. In that study the haplotype HLA-G/01:01:01b/HLA-G/01:01:01 showed a significant higher frequency in control groups and protection against infertility (35). Moreover, the HLA-G UTR-4 haplotype (possessing -964G, -725G, 14 bp del) was associated with a shorter time to achieving pregnancy in an infertility treatment setting when both female and male partners were carriers (36).

One of the conclusions of our study is that polymorphisms in the promoter region of the *HLA-G* gene and the 3’UTR influence the expression and secretion of the soluble form of HLA-G. We found many statistical differences in the sHLA-G concentration in semen between particular haplotype and diplotype carriers. Other studies also indicate the dependence of sHLA-G secretion on its gene polymorphism. Low levels of sHLA-G expression have been associated with HLA-G/01:01:03 and HLA-G/01:05N alleles, intermediate levels are associated with HLA-G/01:01:08 and HLA-G/01:04b alleles, while HLA-G/01:04:01 and HLA-G/01:01g have been known as high secretor alleles (37). A-C-del haplotype is mostly associated with G*01:04/UTR-3 alleles, and this allele has been associated with higher sHLA-G production. A-C-ins haplotype is mostly associated with G*01:01:02, G*01:06, G*01:05, UTR-2, which in turn has been associated with fertility problems (38).

Expression of HLA-G is mainly restricted to the trophoblast cells in the placenta, where it participates in maintaining tolerance at the fetal-maternal interface (39). However, HLA-G can be *de novo* expressed in pathological conditions such as tumors, chronic infections, or after allogeneic transplantation (40, 41). It should be emphasized that HLA-G expression and sHLA-G secretion in different fluids/tissues may depend on various factors, e.g., changes in gene methylation (42, 43) as well as post-transcriptionally by genetic variations in the 3′UTR, which contains several sites for miRNAs binding (41). Moreover, the HLA-G promoter region interacts with specific transcription factors activated by extracellular stimuli induced by hypoxia and heat shock, hormones such as glucocorticoids and progesterone, and cytokines including IL-10 and GM-CSF (41).

Dahl et al. (2014a) correlated sHLA-G levels in seminal plasma with a 14 bp insertion/deletion (ins/del) polymorphism in the 3’UTR of the *HLA-G* gene in couples attending a fertility clinic (13). The concentration of sHLA-G in seminal plasma samples was significantly associated with the HLA-G 14 bp ins/del genotype of the men. Moreover, the del/del genotype showed the highest level of sHLA-G, and the ins/ins genotype showed the lowest level. Considering the combination of the 14 bp insertion with the rs1632947 G allele and the rs1233334 C allele, our results are consistent with the studies by Dahl et al., however in the A-C-ins haplotype (G*01:01:02, G*01:06 or G*01:05N, G010102 promoter, UTR-2), we have the rs1632947 A allele and the opposite is true (13). This haplotype was responsible for the secretion of high levels of sHLA-G. Therefore, the presence of the 14 bp insertion is not always associated with lower sHLA-G secretion. Other polymorphic sites that influence the expression of the *HLA-G* gene are also important, such as rs1632947 and rs1233334. They appear in the promoter region, which are close to regulatory elements and CpG sites, and could alter the binding of transcription factors or promoter methylation and therefore impact the rate of transcription (44). We also reached such conclusions in our previously published studies on sHLA-G tested in blood plasma of women with recurrent implantation failure (17). Significant differences between the levels of sHLA-G in male seminal plasma in relation to HLA-G 3’UTR diplotypes and ins/del HLA-G 14 bp genotypes were also found in the research by Nillson et al. (45). There was a significant difference in semen sHLA-G concentrations between male who were heterozygous for the 14 bp insertion/deletion (del/ins), homozygous for the 14 bp deletion (del/del), or homozygous for the 14 bp insertion (ins/ins) (p = 0.0005). Male homozygous for UTR-2 had significantly lower concentrations of seminal plasma sHLA-G compared with male subjects with the UTR-1/UTR-1 diplotype and the UTR-1/UTR-3 diplotype, respectively. However, they did not find any differences in sHLA-G levels when they compared men with normal sperm parameters and with reduced sperm quality (45).

According to our research, carriers of the A-G-del haplotype and A-C-del/A-G-del diplotype had approximately 3.5 times and 4.9 times (respectively) greater chance of not having children after natural fertilization. Their influence is difficult to explain, because the A-G-del haplotype and the A-C-del/A-G-del diplotype determine a reasonably high sHLA-G secretion, approx. 315 ng/mL in men with normozoospermia. May an excess of sHLA-G be disadvantageous? The HLA-G molecule is considered the immune checkpoint. Its function can therefore be beneficial when expressed by the fetus or transplant and then protects them against rejection, or harmful when expressed by the tumor protecting it from anti-tumor immunity. In fact, higher sHLA-G concentration was associated with progression of several cancers and a poor prognosis of cancer patients (46–48). The upregulation of HLA-G in tumors might be due to proteins associated with inflammation and secreted into the tumor microenvironment. These include IL-6, IL-8 and TNF-α as well as the immune suppressive cytokines IL-10 and TGF-β, which increase HLA-G expression on tumor cells resulting in promotion of evasion from immune cells (49).

Unfortunately, many diplotype analyses cannot be interpreted due to the small number of samples per diplotype, despite the fact that we had nearly 200 semen samples for testing. However, we can comment regarding the secretion of sHLA-G for the most common diplotypes. Normozoospermic men with the A-C-del haplotype and A-C-del/A-C-del diplotype secreted the most sHLA-G into semen (574.1 IU/mL and 1047.0 IU/mL, respectively), while those with the G-C-ins haplotype and G-C-ins/G-C-ins diplotype – the least (80.8 IU/mL and 75.7 IU/mL, respectively). Men with the remaining haplotypes/diplotypes secreted sHLA-G at an intermediate level. Our current studies, as well as data previously published by other researchers (26, 50), provide the evidence of balancing selection acting on the HLA-G promoter, suggesting that the promoters were maintained with high heterozygosity. Thus, divergent HLA-G haplotypes/diplotypes are associated with differential HLA-G expression as was mentioned by Castelli et al. and Rebmann et al. (25, 37). In our study, heterozygous diplotypes account for approximately 74% of all diplotypes. This is probably due to possible better adaptation of people bearing both high and low expression promoters. Heterozygous promoters ensure also the optimal amount of sHLA-G. From an evolutionary point of view, it is beneficial for the male population to have optimal sHLA-G secreted into semen for their reproductive success. On the other hand, the G-C-ins/G-C-ins diplotype amongst men is least beneficial. Chi-square test for trend indicated the insertion allele in this diplotype as being disadvantageous because the odds ratios increased from protective 0.669 in G-C-del/G-C-del men to predisposing 2.396 in G-C-del/G-C-ins men and 3.066 in G-C-ins/G-C-ins men. However, the insertion allele in diplotypes A-C-ins/A-C-del and A-C-ins/A-C-ins was protective, and the odds ratios ranged from 1.497 in A-C-del/A-C-del patients to 0.474 in A-C-ins/A-C-del and to 0.273 in A-C-ins/A-C-ins patients (**Table 2**). The Kruskal-Wallis test confirmed the risk of abnormal sperm parameters from the insertion allele only in the diplotype G-C-ins/G-C-ins, which determined the secretion of a reduced amount of sHLA-G (**Supplementary Table 8**). Unfortunately, in the semen samples we have collected we did not detect A-C-ins/A-C-del and A-C-ins/A-C-ins diplotypes, therefore we could not estimate the level of sHLA-G in these diplotypes.

One of the limitations of our work was the lack of sperm samples from healthy men who have children from natural insemination. Since they have no fertility problems, they have no reason to come to an assisted reproductive clinic and therefore it was not possible to collect material for testing. We could only compare those patients who, despite good sperm parameters, came to the clinic because they cannot have children with their female partners. Moreover, we have only tested sHLA-G once and therefore we could not conclude whether sHLA-G concentration varies in different samples from one male, as was the case in the studies by Nilsson et al. (45). The semen levels of sHLA-G in the samples were fairly consistent over time in the individual males. We had also no data on the exact days of abstinence before the sample was collected for testing, therefore we could not infer the effect of the number of days of sexual abstinence on this concentration.

Nevertheless, in the context of our previously published data on female patients participating in IVF (17), as well as the results currently presented on their partners, *HLA-G* polymorphism and sHLA-G level can affect both female and male infertility.

## Conclusions

We can conclude the following statements:

- Male infertility is associated with *HLA-G* polymorphism.
- Polymorphisms in the *HLA-G* promoter region and 3’UTR influence expression and secretion of its soluble protein.
- Among all *HLA-G* haplotypes and diplotypes, the most unfavourable for male fertility is the G-C-ins haplotype and G-C-ins/G-C-ins diplotype, which determine the secretion of the lowest concentration of the soluble HLA-G molecule.

## Data Availability Statement

The original contributions presented in the study are included in the article/**Supplementary Material**. Further inquiries can be directed to the corresponding author.

## Ethics Statement

The studies involving human participants were reviewed and approved by Local Ethics Committees of University of Wrocław and Polish Mothers’ Memorial Hospital – Research Institute in Łódź. The patients/participants provided their written informed consent to participate in this study.

## Author Contributions

IN conceived and designed the experiments. IN, KP, AT, and AW performed the experiments. IN, KP, AT, and AW analysed the data. PR, MR, JW, AM, and RK contributed to patients and control recruitments. IN and KP wrote the paper. All authors contributed to the article and approved the submitted version.

## Funding

This study was funded by the Polish National Science Centre (grant no. 2014/13/B/NZ5/00273).

## Conflict of Interest

The authors declare that the research was conducted in the absence of any commercial or financial relationships that could be construed as a potential conflict of interest.

## Publisher’s Note

All claims expressed in this article are solely those of the authors and do not necessarily represent those of their affiliated organizations, or those of the publisher, the editors and the reviewers. Any product that may be evaluated in this article, or claim that may be made by its manufacturer, is not guaranteed or endorsed by the publisher.
