# Supplementary material for: The Association of HLA-G Gene Polymorphism and Its Soluble Form With Male Infertility
Source: Front Immunol. 2022 Jan 17;12:791399. doi: 10.3389/fimmu.2021.791399 (PMC8801424; doi:10.3389/fimmu.2021.791399)
Supplement: Supplementary file 1 [file Table_1.docx]

**Supplementary Table 1.** *HLA-G* gene frequencies in fertile men and men who participated in IVF

| **HLA-G** | **Fertile control (%)** | **IVF men**  **(%)** | **IVF men vs. Fertile control** | | |
| --- | --- | --- | --- | --- | --- |
| rs1632947:-964G>A | N = 320 | N = 662 | p | OR | 95% CI |
| AA | 85 (26.56) | 154 (23.26) | 0.267 | 0.838 | 0.61-1.16 |
| AG | 160 (50.00) | 336 (50.76) | 0.838 | 1.031 | 0.78-1.36 |
| GG | 75 (23.44) | 172 (25.98) | 0.433 | 1.146 | 0.83-1.59 |
| Minor allele G | 310 (48.44) | 680 (51.36) | 0.229 | 1.039 | 0.98-1.11 |
| H-W | 0.986 | 0.683 |  |  |  |
| rs1233334:-725G>C/T | N = 319 | N = 663 |  |  |  |
| CC | 217 (68.03) | 462 (69.68) | 0.606 | 1.080 | 0.80-1.45 |
| CG | 79 (24.76) | 160 (24.13) | 0.874 | 0.966 | 0.70-1.34 |
| GG | 7 (2.19) | 11 (1.66) | 0.613 | 0.752 | 0.26-2.31 |
| GT | 2 (0.63) | 2 (0.30) | 0.599 | 0.480 | 0.03-6.65 |
| CT | 14 (4.39) | 28 (4.22) | 0.868 | 0.961 | 0.48-2.00 |
| TT | 0 (0.00) | 0 (0.00) | 1.000 | 0.000 | - |
| Minor allele T | 16 (2.51) | 30 (2.26) | 0.751 | 0.965 | 0.78-1.20 |
| H-W | 0.974 | 0.566 |  |  |  |
| rs371194629: | N = 320 | N = 659 |  |  |  |
| Del/del | 106 (33.13) | 204 (30.96) | 0.510 | 0.905 | 0.67-1.22 |
| Ins/del | 145 (45.31) | 320 (48.56) | 0.375 | 1.139 | 0.86-1.50 |
| Ins/ins | 69 (21.56) | 135 (20.49) | 0.737 | 0.937 | 0.67-1.32 |
| Minor allele ins | 283 (44.22) | 590 (44.76) | 0.846 | 1.007 | 0.95-1.07 |
| H-W | 0.145 | 0.643 |  |  |  |

Values in bold indicate signiﬁcant differences. IVF – *in vitro* fertilization; N – number of patients; p*–*probability; OR – odds ratio; 95% CI – confidence interval from two-sided Fisher’s exact test; H-W – Hardy-Weinberg equilibrium
