# Supplementary material for: The Association of HLA-G Gene Polymorphism and Its Soluble Form With Male Infertility
Source: Front Immunol. 2022 Jan 17;12:791399. doi: 10.3389/fimmu.2021.791399 (PMC8801424; doi:10.3389/fimmu.2021.791399)
Supplement: Supplementary file 2 [file Table_2.docx]

**Supplementary Table 2.** *HLA-G* haplotypes frequencies according to sperm count

| **HLA-G**  **haplotype^*^** | **Normal number of sperm cells (%)** | **Decreased sperm number**  **(%)** | **Moderate OS**  **(%)** | **Severe, very severe OS and AS**  **(%)** | **Decreased sperm number**  **vs. Normal number of sperm cells** | | | **Moderate OS**  **vs. Normal number of sperm cells** | | | **Severe, very severe OS and AS**  **vs. Normal number of sperm cells** | | | **Severe, very severe OS and AS vs. Moderate OS** | | |
| --- | --- | --- | --- | --- | --- | --- | --- | --- | --- | --- | --- | --- | --- | --- | --- | --- |
|  | 2N = 752 | 2N = 488 | 2N = 244 | 2N = 244 | p/p_corr._ | OR | 95% CI | p/p_corr_***_._*** | OR | 95% CI | p/p_corr._ | OR | 95% CI | p/p_corr._ | OR | 95% CI |
| ACdel | 119 (15.82) | 89 (18.24) | 44 (18.03) | 45 (18.44) | 0.277 | 1.186 | 0.87-1.62 | 0.426 | 1.170 | 0.78-1.73 | 0.371 | 1.203 | 0.80-1.78 | 1.000 | 1.028 | 0.63-1.67 |
| ACins | 201 (26.73) | 126 (25.82) | 60 (24.59) | 66 (27.05) | 0.742 | 0.954 | 0.73-1.25 | 0.558 | 0.894 | 0.63-1.26 | 0.934 | 1.016 | 0.72-1.42 | 0.605 | 1.137 | 0.74-1.74 |
| AGdel | 41 (5.45) | 20 (4.10) | 8 (3.28) | 12 (4.92) | 0.347 | 0.741 | 0.41-1.31 | 0.232 | 0.588 | 0.23-1.29 | 0.870 | 0.897 | 0.42-1.78 | 0.494 | 1.525 | 0.56-4.38 |
| ATdel | 4 (0.53) | 4 (0.82) | 1 (0.41) | 3 (1.23) | 0.719 | 1.545 | 0.29-8.34 | 1.000 | 0.770 | 0.02-7.83 | 0.372 | 2.325 | 0.34-13.85 | 0.623 | 3.019 | 0.24-159.31 |
| GCdel | 180 (23.94) | 112 (22.95) | 64 (26.23) | 48 (19.67) | 0.732 | 0.947 | 0.72-1.25 | 0.493 | 1.130 | 0.80-1.59 | 0.188 | 0.778 | 0.53-1.12 | 0.106 | 0.689 | 0.44-1.08 |
| GCins | 124 (16.49) | 80 (16.39) | 40 (16.39) | 40 (16.39) | 1.000 | 0.993 | 0.72-1.36 | 1.000 | 0.993 | 0.65-1.48 | 1.000 | 0.993 | 0.65-1.48 | 1.000 | 1.000 | 0.60-1.66 |
| GGdel | 74 (9.84) | 42 (8.61) | 22 (9.02) | 20 (8.20) | 0.486 | 0.863 | 0.57-1.30 | 0.803 | 0.908 | 0.52-1.52 | 0.529 | 0.818 | 0.46-1.39 | 0.872 | 0.901 | 0.45-1.78 |
| GGins | 2 (0.27) | 1 (0.20) | 0 (0.00) | 1 (0.41) | 1.000 | 0.770 | 0.01-14.83 | 1.000 | 0.000 | 0.00-16.43 | 0.570 | 1.542 | 0.03-29.75 | 1.000 | - | - |
| GTins | 7 (0.93) | 14 (2.87) | 5 (2.05) | 9 (3.69) | **0.013/ns** | 3.140 | 1.18-9.26 | 0.180 | 2.224 | 0.55-8.23 | **0.006/ns** | 4.068 | 1.33-13.01 | 0.417 | 1.828 | 0.54-7.05 |

*Haplotypes were estimated in the following order: rs1632947:-964G>A; rs1233334:-725G>C/T; rs371194629:insATTTGTTCATGCCT/del. Normal number of sperm cells (≥ 15 mln/mL); OS – oligozoospermia; Moderate OS (5-15 mln/mL); Severe OS (1-5 mln/mL); Very severe OS (< 1 mln/mL); AS – azoospermia (lack of sperm cells in ejaculate); Values in bold indicate signiﬁcant differences; N –  number of haplotypes; p*–*probability; p_corr._ – probability after Bonferroni correction for 9 possible haplotypes; OR – odds ratio; 95% CI – confidence interval from two-sided Fisher’s exact test; ns – not significant
