# Supplementary material for: The Association of HLA-G Gene Polymorphism and Its Soluble Form With Male Infertility
Source: Front Immunol. 2022 Jan 17;12:791399. doi: 10.3389/fimmu.2021.791399 (PMC8801424; doi:10.3389/fimmu.2021.791399)
Supplement: Supplementary file 3 [file Table_3.docx]

**Supplementary Table 3.** *HLA-G* haplotypes frequencies according to motility and morphology of sperm cells

| **HLA-G**  **haplotype^*^** | **Normozoospermia**  **(%)** | **Abnormal sperm**  **(%)** | **Asthenozoospermia (%)** | **Teratozoospermia**  **(%)** | **Abnormal sperm**  **vs. Normozoospermia** | | | **Asthenozoospermia**  **vs. Normozoospermia** | | | **Teratozoospermia**  **vs. Normozoospermia** | | |
| --- | --- | --- | --- | --- | --- | --- | --- | --- | --- | --- | --- | --- | --- |
|  | 2N = 536 | 2N = 704 | 2N = 328 | 2N = 252 | p/p_corr._ | OR | 95% CI | p/p_corr._ | OR | 95% CI | p/p_corr_ | OR | 95% CI |
| ACdel | 86 (16.04) | 122 (17,33) | 62 (18.90) | 49 (19.44) | 0.591 | 1.097 | 0.80-1.50 | 0.306 | 1.219 | 0.84-1.77 | 0.265 | 1.263 | 0.84-1.89 |
| ACins | 152 (28.36) | 175 (24,86) | 83 (25.30) | 55 (21.83) | 0.172 | 0.836 | 0.64-1.09 | 0.345 | 0.856 | 0.62-1.18 | 0.056 | 0.706 | 0.49-1.02 |
| AGdel | 30 (5.60) | 31 (4.40) | 17 (5.18) | 11 (4.37) | 0.355 | 0.777 | 0.45-1.35 | 0.878 | 0.922 | 0.47-1.76 | 0.606 | 0.770 | 0.34-1.61 |
| ATdel | 4 (0.75) | 4 (0.57) | 0 (0.00) | 0 (0.00) | 0.732 | 0.760 | 0.14-4.10 | 0.303 | 0.000 | 0.00-2.47 | 0.312 | 0.000 | 0.00-3.22 |
| GCdel | 132 (24.63) | 160 (22.73) | 80 (24.39) | 52 (20.63) | 0.458 | 0.900 | 0.69-1.18 | 1.000 | 0.987 | 0.71-1.37 | 0.241 | 0.796 | 0.54-1.16 |
| GCins | 71 (13.25) | 133 (18.89) | 46 (14.02) | 59 (23.41) | **0.009/ns** | 1.525 | 1.10-2.12 | 0.759 | 1.068 | 0.70-1.62 | **0.0005/0.004** | 2.000 | 1.34-2.99 |
| GGdel | 53 (9.89) | 63 (8.95) | 32 (9.76) | 21 (8.33) | 0.623 | 0.896 | 0.60-1.34 | 1.000 | 0.985 | 0.60-1.60 | 0.516 | 0.829 | 0.46-1.44 |
| GGins | 2 (0.37) | 1 (0.14) | 1 (0.30) | 0 (0.00) | 0.582 | 0.380 | 0.01-7.32 | 1.000 | 0.817 | 0.01-15.75 | 1.000 | 0.000 | 0.00-11.33 |
| GTins | 6 (1.12) | 15 (2.13) | 7 (2.13) | 5 (1.98) | 0.190 | 1.922 | 0.70-6.09 | 0.258 | 1.925 | 0.55-7.00 | 0.342 | 1.787 | 0.43-7.10 |

*Haplotypes were estimated in the following order: rs1632947:-964G>A; rs1233334:-725G>C/T; rs371194629:insATTTGTTCATGCCT/del. Normozoospermia – total number of sperm cells, their concentration, progressive motility and morphology above or equal reference values; Abnormal sperm – at least one parameter of semen below reference value; Asthenozoospermia – number of sperm cells with progressive motility below reference values; Teratozoospermia – number of morphologically normal sperm cells below reference values; N*–*number of haplotypes; p*–* probability; p_corr._ – probability after Bonferroni correction for 9 possible haplotypes; OR – odds ratio; 95% CI – confidence interval from two-sided Fisher’s exact test
