# Supplementary material for: The Association of HLA-G Gene Polymorphism and Its Soluble Form With Male Infertility
Source: Front Immunol. 2022 Jan 17;12:791399. doi: 10.3389/fimmu.2021.791399 (PMC8801424; doi:10.3389/fimmu.2021.791399)
Supplement: Supplementary file 4 [file Table_4.docx]

**Supplementary Table 4.** *HLA-G* diplotypes frequencies according to sperm count

| **HLA-G**  **diplotype^*^** | **Normal number of sperm cells**  **(%)** | **Decreased sperm number**  **(%)** | **Moderate OS**  **(%)** | **Severe,**  **very severe OS and AS (%)** | **Decreased sperm number**  **vs. Normal number of sperm cells** | | | **Moderate OS**  **vs. Normal number of sperm cells** | | | **Severe, very severe OS and AS vs. Normal number of sperm cells** | | | **Severe, very severe OS and AS vs. Moderate OS** | | |
| --- | --- | --- | --- | --- | --- | --- | --- | --- | --- | --- | --- | --- | --- | --- | --- | --- |
|  | N = 376 | N = 244 | N = 122 | N = 122 | p/p_corr_***_._*** | OR | 95% CI | p/p_corr_***_._*** | OR | 95% CI | p/p_corr_***_._*** | OR | 95% CI | p/p_corr_***_._*** | OR | 95% CI |
| ACdel/ACdel | 24 (6.38) | 20 (8.20) | 8 (6.56) | 12 (9.84) | 0.425 | 1.309 | 0.67-2.54 | 1.000 | 1.029 | 0.39-2.45 | 0.227 | 1.598 | 0.70-3.45 | 0.485 | 1.552 | 0.56-4.56 |
| ACdel/AGdel | 31 (8.24) | 14 (5.74) | 8 (6.56) | 6 (4.92) | 0.270 | 0.678 | 0.33-1.35 | 0.699 | 0.781 | 0.30-1.80 | 0.320 | 0.576 | 0.19-1.45 | 0.784 | 0.738 | 0.20-2.51 |
| ACdel/GCdel | 24 (6.38) | 19 (7.79) | 12 (9.84) | 7 (5.74) | 0.520 | 1.238 | 0.63-2.42 | 0.227 | 1.598 | 0.70-3.45 | 1.000 | 0.893 | 0.32-2.21 | 0.340 | 0.559 | 0.18-1.61 |
| ACdel/GGdel | 9 (2.39) | 7 (2.87) | 5 (4.10) | 2 (1.64) | 0.797 | 1.204 | 0.38-3.69 | 0.347 | 1.740 | 0.45-5.92 | 1.000 | 0.680 | 0.07-3.35 | 0.446 | 0.391 | 0.04-2.45 |
| ACins/ACdel | 7 (1.86) | 8 (3.28) | 2 (1.64) | 6 (4.92) | 0.292 | 1.785 | 0.56-5.87 | 1.000 | 0.879 | 0.09-4.70 | 0.096 | 2.720 | 0.74-9.66 | 0.281 | 3.090 | 0.54-31.91 |
| ACins/ACins | 16 (4.26) | 8 (3.28) | 5 (4.10) | 3 (2.46) | 0.671 | 0.763 | 0.28-1.93 | 1.000 | 0.962 | 0.27-2.82 | 0.586 | 0.568 | 0.10-2.03 | 0.722 | 0.591 | 0.09-3.12 |
| ACins/ATdel | 4 (1.06) | 3 (1.23) | 0 (0.00) | 3 (2.46) | 1.000 | 1.157 | 0.17-6.91 | 0.577 | 0.000 | 0.00-4.68 | 0.370 | 2.340 | 0.34-14.04 | 0.247 | - | - |
| ACins/GCdel | 96 (25.53) | 58 (23.77) | 29 (23.77) | 29 (23.77) | 0.636 | 0.910 | 0.61-1.34 | 0.720 | 0.910 | 0.54-1.49 | 0.720 | 0.910 | 0.54-1.49 | 1.000 | 1.000 | 0.53-1.88 |
| AGdel/AGdel | 5 (1.33) | 3 (1.23) | 0 (0.00) | 3 (2.46) | 1.000 | 0.924 | 0.14-4.80 | 0.341 | 0.000 | 0.00-3.37 | 0.412 | 1.868 | 0.29-9.76 | 0.247 | - | - |
| ATdel/ACdel | 0 (0.00) | 1 (0.41) | 1 (0.82) | 0 (0.00) | 0.394 | - | - | 0.245 | - | - | 1.000 | 0.000 | - | 1.000 | 0.000 | 0.00-39.00 |
| GCdel/GCdel | 17 (4.52) | 8 (3.28) | 6 (4.92) | 2 (1.64) | 0.534 | 0.716 | 0.26-1.78 | 0.808 | 1.092 | 0.34-2.99 | 0.182 | 0.352 | 0.04-1.52 | 0.281 | 0.324 | 0.03-1.86 |
| GCdel/GCins | 20 (5.32) | 15 (6.15) | 10 (8.20) | 5 (4.10) | 0.723 | 1.166 | 0.54-2.45 | 0.274 | 1.588 | 0.64-3.68 | 0.812 | 0.761 | 0.22-2.15 | 0.286 | 0.480 | 0.12-1.60 |
| GCins/GCins | 52 (13.83) | 32 (13.11) | 15 (12.30) | 17 (13.93) | 0.904 | 0.941 | 0.57-1.54 | 0.761 | 0.874 | 0.44-1.65 | 1.000 | 1.009 | 0.52-1.87 | 0.850 | 1.154 | 0.51-2.62 |
| GCins/ACins | 0 (0.00) | 1 (0.41) | 0 (0.00) | 1 (0.82) | 0.394 | - | - | 1.000 | 0.000 | - | 0.245 | - | - | 1.000 | - | - |
| GGdel/ACins | 57 (15.16) | 28 (11.48) | 14 (11.48) | 14 (11.48) | 0.232 | 0.726 | 0.43-1.20 | 0.372 | 0.726 | 0.36-1.38 | 0.372 | 0.726 | 0.36-1.38 | 1.000 | 1.000 | 0.42-2.38 |
| GGdel/GCdel | 5 (1.33) | 3 (1.23) | 1 (0.82) | 2 (1.64) | 1.000 | 0.924 | 0.14-4.80 | 1.000 | 0.614 | 0.01-5.56 | 0.682 | 1.236 | 0.12-7.67 | 1.000 | 2.011 | 0.10-119.86 |
| GGdel/GGdel | 1 (0.27) | 2 (0.82) | 1 (0.82) | 1 (0.82) | 0.565 | 3.093 | 0.16-183.12 | 0.430 | 3.090 | 0.04-243.43 | 0.430 | 3.090 | 0.04-243.43 | 1.000 | 1.000 | 0.01-79.09 |
| GGdel/GGins | 1 (0.27) | 0 (0.00) | 0 (0.00) | 0 (0.00) | 1.000 | 0.000 | 0.00-60.04 | 1.000 | 0.000 | 0.00-119.97 | 1.000 | 0.000 | 0.00-119.97 | 1.000 | 0.000 | - |
| GTins/ACins | 5 (1.33) | 12 (4.92) | 5 (4.10) | 7 (5.74) | **0.011/ns** | 3.829 | 1.24-14.06 | 0.070 | 3.162 | 0.71-13.99 | **0.012/ns** | 4.500 | 1.20-18.35 | 0.769 | 1.422 | 0.38-5.86 |
| GTins/GCdel | 1 (0.27) | 1 (0.41) | 0 (0.00) | 1 (0.82) | 1.000 | 1.542 | 0.02-121.34 | 1.000 | 0.000 | 0.00-119.97 | 0.430 | 3.090 | 0.04-243.43 | 1.000 | - | - |
| GGins/GTins | 1 (0.27) | 1 (0.41) | 0 (0.00) | 1 (0.82) | 1.000 | 1.542 | 0.02-121.34 | 1.000 | 0.000 | 0.00-119.97 | 0.430 | 3.090 | 0.04-243.43 | 1.000 | - | - |

^*^Diplotypes were estimated in the following order: rs1632947:-964G>A; rs1233334:-725G>C/T; rs371194629:insATTTGTTCATGCCT/del. Normal number of sperm cells (≥ 15 mln/mL); OS – oligozoospermia; Moderate OS (5-15 mln/mL); Severe OS (1-5 mln/mL); Very severe OS (< 1 mln/mL); AS – azoospermia (lack of sperm cells in ejaculate); Values in bold indicate signiﬁcant differences; N *–*number of diplotypes; p*–*probability; p_corr._ – probability after Bonferroni correction for 23 possible diplotypes; OR – odds ratio; 95% CI – confidence interval from two-sided Fisher’s exact test; ns – not significant
