# Supplementary material for: The Association of HLA-G Gene Polymorphism and Its Soluble Form With Male Infertility
Source: Front Immunol. 2022 Jan 17;12:791399. doi: 10.3389/fimmu.2021.791399 (PMC8801424; doi:10.3389/fimmu.2021.791399)
Supplement: Supplementary file 5 [file Table_5.docx]

**Supplementary Table 5.** *HLA-G* diplotypes frequencies according to motility and morphology of sperm cells

| **HLA-G**  **diplotype*** | **Normozoospermia (%)** | **Abnormal sperm (%)** | **Asthenozoospermia (%)** | **Teratozoospermia**  **(%)** | **Abnormal sperm**  **vs. Normozoospermia** | | | **Asthenozoospermia**  **vs. Normozoospermia** | | | **Teratozoospermia**  **vs. Normozoospermia** | | |
| --- | --- | --- | --- | --- | --- | --- | --- | --- | --- | --- | --- | --- | --- |
|  | N = 268 | N = 352 | N = 164 | N = 126 | p/p_corr._ | OR | 95% CI | p/p_corr._ | OR | 95% CI | p/p_corr._ | OR | 95% CI |
| ACdel/ACdel | 17 (6.34) | 27 (7.67) | 13 (7.93) | 12 (9.52) | 0.636 | 1.226 | 0.63-2.45 | 0.562 | 1.270 | 0.55-2.87 | 0.301 | 1.552 | 0.65-3.58 |
| ACdel/AGdel | 22 (8.21) | 23 (6.53) | 13 (7.93) | 9 (7.14) | 0.439 | 0.782 | 0.41-1.51 | 1.000 | 0.963 | 0.43-2.07 | 0.842 | 0.860 | 0.34-2.02 |
| ACdel/GCdel | 18 (6.72) | 25 (7.10) | 12 (7.32) | 10 (7.94) | 0.875 | 1.062 | 0.54-2.12 | 0.847 | 1.096 | 0.47-2.48 | 0.677 | 1.197 | 0.48-2.84 |
| ACdel/GGdel | 7 (2.61) | 9 (2.56) | 4 (2.44) | 5 (3.97) | 1.000 | 0.978 | 0.32-3.13 | 1.000 | 0.932 | 0.20-3.74 | 0.533 | 1.539 | 0.38-5.76 |
| ACins/ACdel | 5 (1.87) | 10 (2.84) | 7 (4.27) | 1 (0.79) | 0.600 | 1.537 | 0.47-5.80 | 0.226 | 2.340 | 0.63-9.52 | 0.669 | 0.422 | 0.01-3.82 |
| ACins/ACins | 15 (5.60) | 9 (2.56) | 3 (1.83) | 1 (0.79) | 0.060 | 0.443 | 0.17-1.10 | 0.080 | 0.315 | 0.06-1.14 | **0.026/ns** | 0.135 | 0.00-0.90 |
| ACins/ATdel | 4 (1.49) | 3 (0.85) | 0 (0.00) | 0 (0.00) | 0.473 | 0.568 | 0.08-3.39 | 0.302 | 0.000 | 0.00-2.47 | 0.311 | 0.000 | 0.00-3.22 |
| ACins/GCdel | 69 (25.75) | 85 (24.15) | 44 (26.83) | 32 (25.40) | 0.708 | 0.918 | 0.63-1.35 | 0.822 | 1.057 | 0.66-1.68 | 1.000 | 0.982 | 0.58-1.63 |
| AGdel/AGdel | 4 (1.49) | 4 (1.14) | 2 (1.22) | 1 (0.79) | 0.732 | 0.759 | 0.14-4.11 | 1.000 | 0.815 | 0.07-5.76 | 1.000 | 0.529 | 0.01-5.42 |
| ATdel/ACdel | 0 (0.00) | 1 (0.28) | 0 (0.00) | 0 (0.00) | 1.000 | - | - | 1.000 | 0.000 | - | 1.000 | 0.000 | - |
| GCdel/GCdel | 13 (4.85) | 12 (3.41) | 6 (3.66) | 1 (0.79) | 0.413 | 0.693 | 0.28-1.68 | 0.636 | 0.745 | 0.23-2.15 | **0.044/ns** | 0.157 | 0.00-1.07 |
| GCdel/GCins | 15 (5.60) | 20 (5.68) | 6 (3.66) | 7 (5.56) | 1.000 | 1.016 | 0.48-2.18 | 0.490 | 0.641 | 0.20-1.79 | 1.000 | 0.992 | 0.33-2.67 |
| GCins/GCins | 28 (10.45) | 56 (15.91) | 20 (12.20) | 26 (20.63) | 0.058 | 1.620 | 0.98-2.74 | 0.637 | 1.190 | 0.61-2.28 | **0.008/ns** | 2.224 | 1.19-4.15 |
| GCins/ACins | 0 (0.00) | 1 (0.28) | 0 (0.00) | 0 (0.00) | 1.000 | - | - | 1.000 | 0.000 | - | 1.000 | 0.000 | - |
| GGdel/ACins | 39 (14.55) | 46 (13.07) | 22 (13.41) | 15 (11.90) | 0.638 | 0.883 | 0.54-1.44 | 0.778 | 0.910 | 0.49-1.65 | 0.532 | 0.794 | 0.39-1.55 |
| GGdel/GCdel | 4 (1.49) | 4 (1.14) | 4 (2.44) | 1 (0.79) | 0.732 | 0.759 | 0.14-4.11 | 0.485 | 1.648 | 0.30-8.98 | 1.000 | 0.529 | 0.01-5.42 |
| GGdel/GGdel | 1 (0.37) | 2 (0.57) | 1 (0.61) | 0 (0.00) | 1.000 | 1.525 | 0.08-90.30 | 1.000 | 1.636 | 0.02-128.93 | 1.000 | 0.000 | 0.00-82.85 |
| GGdel/GGins | 1 (0.37) | 0 (0.00) | 0 (0.00) | 0 (0.00) | 0.432 | 0.000 | 0.00-29.69 | 1.000 | 0.000 | 0.00-63.67 | 1.000 | 0.000 | 0.00-82.85 |
| GTins/ACins | 5 (1.87) | 12 (3.41) | 4 (2.44) | 5 (3.97) | 0.323 | 1.855 | 0.60-6.81 | 0.736 | 1.314 | 0.26-6.20 | 0.301 | 2.169 | 0.49-9.62 |
| GTins/GCdel | 0 (0.00) | 2 (0.57) | 2 (1.22) | 0 (0.00) | 0.508 | - | - | 0.144 | - | - | 1.000 | 0.000 | - |
| GGins/GTins | 1 (0.37) | 1 (0.28) | 1 (0.61) | 0 (0.00) | 1.000 | 0.761 | 0.01-59.91 | 1.000 | 1.636 | 0.02-128.93 | 1.000 | 0.000 | 0.00-82.85 |

*Diplotypes were estimated in the following order: rs1632947:-964G>A; rs1233334:-725G>C/T; rs371194629:insATTTGTTCATGCCT/del. Normozoospermia – total number of sperm cells, their concentration, progressive motility and morphology above or equal reference values; Abnormal sperm – at least one parameter of semen below reference value; Asthenozoospermia – number of sperm cells with progressive motility below reference values; Teratozoospermia – number of morphologically normal sperm cells below reference values; N – number of diplotypes; p*–* probability; p_corr._ – probability after Bonferroni correction for 23 possible diplotypes; OR – odds ratio; 95% CI – confidence interval from two-sided Fisher’s exact test
