# Supplementary material for: The Association of HLA-G Gene Polymorphism and Its Soluble Form With Male Infertility
Source: Front Immunol. 2022 Jan 17;12:791399. doi: 10.3389/fimmu.2021.791399 (PMC8801424; doi:10.3389/fimmu.2021.791399)
Supplement: Supplementary file 6 [file Table_6.docx]

**Supplementary Table 6.** Statistical analysis of sHLA-G secretion in men participating in IVF

|  | **Normozoospermia** | **Abnormal sperm** | **Asthenozospermia** | **Teratozoospermia** | **Normal number of sperm cells** | **Moderate OS** | **Severe, very severe OS, AS** |
| --- | --- | --- | --- | --- | --- | --- | --- |
| Number of patients | 60 | 123 | 40 | 95 | 121 | 33 | 29 |
| Minimum | 22.0 | 15.0 | 26.6 | 15.0 | 22.0 | 15.0 | 26.6 |
| 25% Percentile | 85.1 | 78.5 | 81.8 | 72.6 | 78.0 | 99.8 | 85.4 |
| Median | 288.9 | 227.9 | 261.7 | 201.3 | 259.7 | 196.3 | 258.3 |
| 75% Percentile | 574.9 | 594.9 | 781.8 | 678.1 | 648.8 | 352.0 | 683.0 |
| Maximum | 1260.0 | 1260.0 | 1260.0 | 1260.0 | 1260.0 | 1260.0 | 1260.0 |
| Mean | 418.8 | 385.9 | 457.3 | 398.0 | 413.9 | 322.3 | 409.7 |
| Std. deviation | 390.6 | 394.0 | 436.5 | 425.4 | 405.8 | 356.4 | 374.7 |
| Std. error | 50.4 | 35.5 | 69.0 | 43.7 | 36.9 | 62.0 | 69.6 |
| Lower 95% CI of mean | 318.0 | 315.6 | 317.7 | 311.3 | 340.8 | 195.9 | 267.2 |
| Upper 95% CI of mean | 519.7 | 456.3 | 596.9 | 484.6 | 486.9 | 448.6 | 552.3 |
| D'Agostino & Pearson  omnibus normality test K^2^ | 9.0 | 21.7 | 6.2 | 15.3 | 17.1 | 18.9 | 4.9 |

Normozoospermia – total number of sperm cells, their concentration, progressive motility and morphology above or equal reference values; Abnormal sperm – at least one parameter of semen below reference value; Asthenozoospermia – number of sperm cells with progressive motility below reference values; Teratozoospermia – number of morphologically normal sperm cells below reference values; Normal number of sperm cells – ≥ 15 mln/mL; OS – oligozoospermia; Moderate OS (5-15 mln/mL); Severe OS (1-5 mln/mL); Very severe OS (< 1 mln/mL); AS – azoospermia (lack of sperm cells in ejaculate);
