# Supplementary material for: The Association of HLA-G Gene Polymorphism and Its Soluble Form With Male Infertility
Source: Front Immunol. 2022 Jan 17;12:791399. doi: 10.3389/fimmu.2021.791399 (PMC8801424; doi:10.3389/fimmu.2021.791399)
Supplement: Supplementary file 7 [file Table_7.docx]

**Supplementary Table 7.** HLA-G value (IU/ml) in sperm depending on particular HLA-G haplotypes in normozoospermic men and men with abnormal sperm parameters

| **Haplotype*** | **Number of patients** | **Minimum** | **25% Percentile** | **Median** | **75% Percentile** | **Maximum** | **Mean** | **Std. Deviation** | **Std. Error** | **Lower 95% CI of mean** | **Upper 95% CI of mean** | **D'Agostino & Pearson omnibus normality test K2** |
| --- | --- | --- | --- | --- | --- | --- | --- | --- | --- | --- | --- | --- |
| **Normozoospermia** | | | | | | | | | | | | |
| ACdel | 22 | 171.3 | 303.4 | **574.1 ^a, b, c^** | 1180.0 | 1260.0 | 666.2 | 410.7 | 87.6 | 484.1 | 848.3 | 7.4 |
| ACins | 23 | 31.3 | 145.9 | **391.2 ^d^** | 920.5 | 1260.0 | 519.2 | 423.3 | 88.3 | 336.2 | 702.3 | 4.4 |
| AGdel | 12 | 128.3 | 193.8 | **315.2 ^e^** | 639.7 | 1036.0 | 431.9 | 321.9 | 92.9 | 227.4 | 636.4 | 3.8 |
| ATdel | 2 | 469.4 | 469.4 | 847.1 | 1225.0 | 1225.0 | 847.1 | 534.2 | 377.8 | -3953.0 | 5647.0 | N too small |
| GCdel | 25 | 52.5 | 148.7 | **347.7 ^f^** | 800.3 | 1260.0 | 472.7 | 384.8 | 77.0 | 313.9 | 631.5 | 3.3 |
| GCins | 28 | 22.0 | 54.6 | **80.8 ^g, h^** | 125.3 | 498.3 | 120.8 | 121.4 | 23.0 | 73.7 | 167.9 | 26.3 |
| GGdel | 7 | 46.2 | 52.6 | 145.9 | 391.2 | 476.9 | 221.4 | 186.1 | 70.4 | 49.3 | 393.5 | N too small |
| GTins | 1 | 31.3 | 31.3 | 31.3 | 31.3 | 31.3 | 31.3 | 0.0 | 0.0 | 0.0 | 0.0 | N too smal |
| **Abnormal sperm parameters** | | | | | | | | | | | | |
| ACdel | 47 | 132.7 | 365.4 | **650.0 ^i, j, k, l, m^** | 1228.0 | 1260.0 | 688.6 | 392.0 | 57.2 | 573.5 | 803.7 | 16.9 |
| ACins | 50 | 31.7 | 97.3 | **261.7 ^n, o^** | 772.5 | 1260.0 | 455.1 | 425.7 | 60.2 | 334.2 | 576.1 | 7.9 |
| AGdel | 5 | 132.7 | 233.4 | **395.3 ^p, q^** | 744.3 | 747.9 | 470.1 | 268.5 | 120.1 | 136.8 | 803.5 | N too small |
| ATdel | 1 | 594.9 | 594.9 | 594.9 | 594.9 | 594.9 | 594.9 | 0.0 | 0.0 | 0.0 | 0.0 | N too smal |
| GCdel | 47 | 45.7 | 150.6 | **351.5 ^r, s^** | 886.9 | 1260.0 | 509.8 | 432.5 | 63.1 | 382.8 | 636.8 | 10.1 |
| GCins | 70 | 15.0 | 35.1 | **67.5 ^t^** | 125.5 | 461.0 | 102.3 | 93.4 | 11.2 | 80.0 | 124.5 | 28.3 |
| GGdel | 21 | 31.7 | 69.5 | 200.7 | 255.7 | 518.7 | 190.4 | 140.7 | 30.7 | 126.3 | 254.4 | 3.8 |
| GTins | 3 | 79.7 | 79.7 | 178.8 | 258.3 | 258.3 | 172.3 | 89.5 | 51.7 | -50.0 | 394.5 | N too small |
| **Asthenozoospermia** | | | | | | | | | | | | |
| ACdel | 21 | 395.3 | 441.5 | **820.2 ^u, v, w^** | 1228.0 | 1260.0 | 808.4 | 336.9 | 73.5 | 655.0 | 961.8 | 7.0 |
| ACins | 15 | 31.7 | 90.0 | **246.4 ^x^** | 995.6 | 1260.0 | 457.2 | 476.9 | 123.1 | 193.1 | 721.3 | 3.6 |
| AGdel | 1 | 395.3 | 395.3 | 395.3 | 395.3 | 395.3 | 395.3 | 0.0 | 0.0 | 0.0 | 0.0 | N too smal |
| GCdel | 12 | 77.4 | 109.8 | **475.8 ^y^** | 1212.0 | 1260.0 | 620.4 | 507.3 | 146.5 | 298.0 | 942.7 | 6.8 |
| GCins | 20 | 26.6 | 31.8 | **53.0 ^z^** | 88.0 | 461.0 | 94.9 | 109.4 | 24.5 | 43.7 | 146.1 | 24.2 |
| GGdel | 7 | 31.7 | 90.0 | 209.9 | 265.0 | 481.8 | 205.1 | 149.5 | 56.5 | 66.9 | 343.4 | N too small |
| GTins | 2 | 79.7 | 79.7 | 169.0 | 258.3 | 258.3 | 169.0 | 126.3 | 89.3 | -965.6 | 1304.0 | N too small |
| **Teratozoospermia** | | | | | | | | | | | | |
| ACdel | 39 | 132.7 | 365.4 | **666.6 ^aa, ab,^ ^ac, ad^** | 1255.0 | 1260.0 | 720.3 | 411.5 | 65.9 | 587.0 | 853.7 | 25.3 |
| ACins | 37 | 39.4 | 87.8 | **233.1 ^ae^** | 898.5 | 1260.0 | 479.9 | 464.5 | 76.4 | 325.0 | 634.8 | 8.5 |
| AGdel | 5 | 132.7 | 233.4 | 395.3 **^af, ag^** | 744.3 | 747.9 | 470.1 | 268.5 | 120.1 | 136.8 | 803.5 | N too small |
| GCdel | 35 | 45.7 | 166.0 | 365.4 **^ah, ai^** | 995.6 | 1260.0 | 558.8 | 460.9 | 77.9 | 400.5 | 717.1 | 16.3 |
| GCins | 55 | 15.0 | 31.8 | 59.2 **^aj, ak^** | 113.9 | 291.6 | 80.5 | 71.8 | 9.7 | 61.1 | 99.9 | 26.0 |
| GGdel | 16 | 39.4 | 66.4 | 105.4 | 241.0 | 518.7 | 182.2 | 150.8 | 37.7 | 101.8 | 262.5 | 5.3 |
| GTins | 3 | 79.7 | 79.7 | 178.8 | 258.3 | 258.3 | 172.3 | 89.5 | 51.7 | -50.0 | 394.5 | N too small |

*Haplotypes were estimated in the following order: rs1632947:-964G>A; rs1233334:-725G>C/T; rs371194629:insATTTGTTCATGCCT/del. Normozoospermia – total number of sperm cells, their concentration, progressive motility and morphology above or equal reference values; Abnormal sperm – at least one parameter of semen below reference value; Asthenzooospermia – number of sperm cells with progressive motility below reference values; Teratozoospermia – number of morphologically normal sperm cells below reference values; Values in bold indicate signiﬁcant differences; p*–*probability calculated by Mann-Whitney test or t-test; OR – odds ratio; 95% CI – confidence interval from two-sided Fisher’s exact test

**Normozoospermic men vs. normozoospermic men**: ^a^p = 0.051 ACdel vs GCdel; ^b^p < 0.0001 ACdel vs GCins; ^c^p = 0.009 ACdel vs GGdel; ^d^p = 0.0004 ACins vs GCins; ^e^p < 0.0001 AGdel vs GCins; ^f^p < 0.0001 GCdel vs GCins;

**Normozoospermic men vs. teratozoospermic men**: ^g^p = 0.009 GCins vs GCins;

**Normozoospermic men vs. astenozoospermic men:** ^h^p = 0.058 GCins vs GCins;

**Men with abnormal sperm vs. men with abnormal sperm:** ^i^p = 0.001 ACdel vs ACins; ^j^p = 0.010 ACdel vs GCdel; ^k^p < 0.0001 ACdel vs GCins; ^l^p < 0.0001 ACdel vs GGdel; ^m^p = 0.014 ACdel vs GTins; ^n^p < 0.0001 ACins GCins; ^o^p = 0.023 ACins vs GGdel; ^p^p = 0.001 AGdel vs GCins; ^q^p = 0.016 AGdel vs GGdel; ^r^p < 0.0001 GCdel vs GCins; ^s^p = 0.005 GCdel vs GGdel; ^t^p = 0.004 GCins vs GGdel;

**Astenozoospermic men vs. astenozoospermic men**: ^u^p = 0.010 ACdel vs ACins; ^v^p < 0.0001 ACdel vs GCins; ^w^p = 0.0003 ACdel vs GGdel; ^x^p = 0.0006 ACins vs GCins; ^y^p = 0.0001 GCdel vs GCins; ^z^p = 0.0329 GCins vs GGdel;

**Teratozoospermic men vs. teratozoospermic men**: ^aa^p = 0.006 ACdel vs ACins; ^ab^p < 0.0001 ACdel vs GCins; ^ac^p < 0.0001 ACdel vs GGdel; ^ad^p = 0.014 ACdel vs GTins; ^ae^p < 0.0001 ACins vs GCins; ^af^p = 0.001 AGdel vs GCins; ^ag^p = 0.019 AGdel vs GGdel; ^ah^p < 0.0001 GCdel vs GCins; ^ai^p = 0.007 GCdel vs GGdel; ^aj^p = 0.002 GCins vs GGdel; ^ak^p = 0.042 GCins vs GTins;
