# Supplementary material for: The Association of HLA-G Gene Polymorphism and Its Soluble Form With Male Infertility
Source: Front Immunol. 2022 Jan 17;12:791399. doi: 10.3389/fimmu.2021.791399 (PMC8801424; doi:10.3389/fimmu.2021.791399)
Supplement: Supplementary file 8 [file Table_8.docx]

**Supplementary Table 8.** HLA-G value (IU/ml) in sperm depending on particular HLA-G diplotypes in normozoospermic men and men with abnormal sperm parameters

| **Diplotype** | **Number of patients** | **Minimum** | **25% Percentile** | **Median** | **75% Percentile** | **Maximum** | **Mean** | **Std. Deviation** | **Std. Error** | **Lower 95% CI**  **of mean** | **Upper 95% CI**  **of mean** | **D'Agostino & Pearson omnibus normality test K^2^** |
| --- | --- | --- | --- | --- | --- | --- | --- | --- | --- | --- | --- | --- |
| **Normozoospermia** | | | | | | | | | | | | |
| ACdel/ACdel | 6 | 173.9 | 475.3 | **1047.0 ^a, b, c, d^** | 1260.0 | 1260.0 | 893.8 | 440.5 | 179.8 | 431.6 | 1356.0 | N too small |
| ACdel/AGdel | 8 | 171.3 | 268.7 | **315.2 ^e^** | 433.4 | 697.9 | 356.8 | 160.3 | 56.7 | 222.8 | 490.9 | 7.0 |
| ACdel/GCdel | 2 | 503.2 | 503.2 | 537.9 | 572.5 | 572.5 | 537.9 | 49.0 | 34.7 | 97.5 | 978.2 | N too small |
| ACins/ATdel | 2 | 469.4 | 469.4 | 847.1 | 1225.0 | 1225.0 | 847.1 | 534.2 | 377.8 | -3953.0 | 5647.0 | N too small |
| ACins/GCdel | 13 | 61.3 | 260.7 | **720.1 ^f, g, h^** | 1049.0 | 1260.0 | 666.7 | 417.6 | 115.8 | 414.3 | 919.0 | 4.4 |
| AGdel/AGdel | 2 | 128.3 | 128.3 | 582.2 | 1036.0 | 1036.0 | 582.2 | 641.9 | 453.9 | -5185.0 | 6350.0 | N too small |
| GCdel/GCdel | 1 | 160.6 | 160.6 | 160.6 | 160.6 | 160.6 | 160.6 | 0.0 | 0.0 | 0.0 | 0.0 | N too small |
| GCdel/GCins | 8 | 52.5 | 76.8 | **112.5 ^i^** | 444.1 | 498.3 | 219.2 | 190.0 | 67.2 | 60.4 | 378.1 | 2.5 |
| GCins/GCins | 10 | 22.0 | 48.2 | 75.7 | 101.5 | 181.9 | 81.5 | 45.4 | 14.3 | 49.0 | 113.9 | 4.9 |
| GGdel/ACins | 7 | 46.2 | 52.6 | 145.9 | 391.2 | 476.9 | 221.4 | 186.1 | 70.4 | 49.3 | 393.5 | N too small |
| GTins/ACins | 1 | 31.3 | 31.3 | 31.3 | 31.3 | 31.3 | 31.3 | 0.0 | 0.0 | 0.0 | 0.0 | N too small |
| **Abnormal sperm parameters** | | | | | | | | | | | | |
| ACdel/ACdel | 16 | 236.9 | 405.5 | **743.4 ^j, k, l, m^** | 1259.0 | 1260.0 | 802.9 | 393.2 | 98.3 | 593.4 | 1012.0 | 8.0 |
| ACdel/AGdel | 5 | 132.7 | 233.4 | **395.3 ^n, o^** | 744.3 | 747.9 | 470.1 | 268.5 | 120.1 | 136.8 | 803.5 | N too small |
| ACdel/GCdel | 6 | 150.6 | 184.8 | **459.4 ^p, q^** | 800.0 | 1069.0 | 507.5 | 374.3 | 152.8 | 114.7 | 900.3 | N too small |
| ACdel/GGdel | 4 | 227.9 | 229.8 | **282.8 ^r, s^** | 443.9 | 481.8 | 318.8 | 118.2 | 59.1 | 130.7 | 506.9 | N too small |
| ACins/ATdel | 1 | 594.9 | 594.9 | 594.9 | 594.9 | 594.9 | 594.9 | 0.0 | 0.0 | 0.0 | 0.0 | N too small |
| ACins/GCdel | 29 | 45.7 | 228.0 | **495.9 ^t, u^** | 1194.0 | 1260.0 | 652.5 | 452.0 | 83.9 | 480.5 | 824.4 | 17.2 |
| GCdel/GCdel | 1 | 91.1 | 91.1 | **91.1 ^v^** | 91.1 | 91.1 | 91.1 | 0.0 | 0.0 | 0.0 | 0.0 | N too small |
| GCdel/GCins | 10 | 46.5 | 75.1 | 149.7 | 249.5 | 461.0 | 180.9 | 134.0 | 42.4 | 85.1 | 276.8 | 3.8 |
| GCins/GCins | 30 | 15.0 | 32.1 | **59.6^w^** | 116.7 | 291.6 | 89.2 | 79.6 | 14.5 | 59.5 | 118.9 | 10.6 |
| GGdel/ACins | 17 | 31.7 | 61.1 | 99.8 | 244.6 | 518.7 | 160.1 | 130.5 | 31.6 | 93.1 | 227.2 | 9.1 |
| GTins/ACins | 3 | 79.7 | 79.7 | 178.8 | 258.3 | 258.3 | 172.3 | 89.5 | 51.7 | -50.0 | 394.5 | N too small |
| **Asthenozoospermia** | | | | | | | | | | | | |
| ACdel/ACdel | 9 | 399.3 | 525.6 | **820.2 ^x, y^** | 1244.0 | 1260.0 | 835.0 | 345.7 | 115.2 | 569.3 | 1101.0 | 1.723 |
| ACdel/AGdel | 1 | 395.3 | 395.3 | **395.3 ^z, aa^** | 395.3 | 395.3 | 395.3 | 0.0 | 0.0 | 0.0 | 0.0 | N too small |
| ACdel/GCdel | 1 | 1069.0 | 1069.0 | 1069.0 | 1069.0 | 1069.0 | 1069.0 | 0.0 | 0.0 | 0.0 | 0.0 | N too small |
| ACdel/GGdel | 1 | 481.8 | 481.8 | 481.8 | 481.8 | 481.8 | 481.8 | 0.0 | 0.0 | 0.0 | 0.0 | N too small |
| ACins/GCdel | 7 | 77.36 | 222.9 | 995.6 | 1260.0 | 1260.0 | 795.2 | 520.3 | 196.6 | 314.0 | 1276.0 | N too small |
| GCdel/GCdel | 1 | 91.11 | 91.11 | 91.11 | 91.11 | 91.11 | 91.11 | 0.0 | 0.0 | 0.0 | 0.0 |  |
| GCdel/GCins | 2 | 166.0 | 166.0 | 313.5 | 461.0 | 461.0 | 313.5 | 208.6 | 147.5 | -1561.0 | 2188.0 | N too small |
| GCins/GCins | 9 | 26.62 | 31.39 | 45.99 | 80.3 | 247.4 | 70.62 | 69.6 | 23.2 | 17.12 | 124.1 | 19.14 |
| GGdel/ACins | 6 | 31.65 | 75.44 | 160.4 | 251.0 | 265.0 | 159.0 | 94.61 | 38.62 | 59.71 | 258.3 | N too small |
| GTins/ACins | 2 | 79.74 | 79.74 | 169.0 | 258.3 | 258.3 | 169.0 | 126.3 | 89.29 | -965.6 | 1304.0 | N too small |
| **Teratozoospermia** | | | | | | | | | | | | |
| ACdel/ACdel | 12 | 365.4 | 434.8 | **1024.0 ^ab, ac, ad, ae, af, ag^** | 1260.0 | 1260.0 | 892.6 | 396.6 | 114.5 | 640.6 | 1145.0 | 7.715 |
| ACdel/AGdel | 5 | 132.7 | 233.4 | **395.3 ^ah, ai, aj^** | 744.3 | 747.9 | 470.1 | 268.5 | 120.1 | 136.8 | 803.5 | N too small |
| ACdel/GCdel | 6 | 150.6 | 184.8 | **459.4 ^ak, al^** | 800.0 | 1069.0 | 507.5 | 374.3 | 152.8 | 114.7 | 900.3 | N too small |
| ACdel/GGdel | 4 | 227.9 | 229.8 | **282.8 ^am, an, ao^** | 443.9 | 481.8 | 318.8 | 118.2 | 59.1 | 130.7 | 506.9 | N too small |
| ACins/GCdel | 22 | 45.71 | 211.9 | **776.9 ^ap, aq ar^** | 1260.0 | 1260.0 | 709.1 | 473.0 | 100.8 | 499.4 | 918.9 | 14.2 |
| GCdel/GCins | 7 | 46.48 | 74.27 | **133.3 ^as^** | 201.3 | 215.5 | 130.3 | 66.79 | 25.25 | 68.56 | 192.1 | N too small |
| GCins/GCins | 24 | 15.0 | 31.18 | **46.21 ^at, au^** | 77.03 | 291.6 | 73.23 | 70.91 | 14.48 | 43.28 | 103.2 | 23.86 |
| GGdel/ACins | 12 | 39.35 | 59.95 | 86.06 | 178.3 | 518.7 | 136.6 | 134.6 | 38.87 | 51.07 | 222.2 | 19.93 |
| GTins/ACins | 3 | 79.74 | 79.74 | 178.8 | 258.3 | 258.3 | 172.3 | 89.47 | 51.66 | -49.98 | 394.5 | N too small |

*Diplotypes were estimated in the following order: rs1632947:-964G>A; rs1233334:-725G>C/T; rs371194629:insATTTGTTCATGCCT/del. Normozoospermia – total number of sperm cells, their concentration, progressive motility and morphology above or equal reference values; Abnormal sperm – at least one parameter of semen below reference value; Values in bold indicate signiﬁcant differences; p*–*probability calculated by Mann-Whitney test or t-test; OR – odds ratio; 95% CI – confidence interval from two-sided Fisher’s exact test

**Normozoospermic men vs normozoospermic men:** ^a^p = 0.045 ACdel/ACdel vs ACdel/AGdel; ^b^p = 0.008 ACdel/ACdel vs GCdel/GCins; ^c^p = 0.002 ACdel/ACdel vs GCins/GCins; ^d^p = 0.012 ACdel/ACdel vs GGdel/ACins; ^e^p < 0.0001 ACdel/AGdel vs GCins/GCins; ^f^p = 0.011 ACins/GCdel vs GCdel/GCins; ^g^p = 0.0003 ACins/GCdel vs GCins/GCins; ^h^p = 0.027 ACins/GCdel vs GGdel/ACins; ^i^p = 0.040 GCdel/GCins vs GCins/GCins;

**Men with abnormal sperm vs men with abnormal sperm:** ^j^p = 0.016 ACdel/ACdel vs ACdel/GGdel; ^k^p < 0.0001 ACdel/ACdel vs GCins/GCins; ^l^p < 0.0001 ACdel/ACdel vs GGdel/ACins; ^m^p = 0.012 ACdel/ACdel vs GTins/ACins; ^n^p = 0.001 ACdel/AGdel vs GCins/GCins; ^o^p = 0.010 ACdel/AGdel vs GGdel/ACins; ^p^p = 0.001 ACdel/GCdel vs GCins/GCins; ^q^p = 0.027 ACdel/GCdel vs GGdel/ACins; ^r^p = 0.004 ACdel/GGdel vs GCins/GCins; ^s^p = 0.054 ACdel/GGdel vs GGdel/ACins; ^t^p < 0.0001 ACins/GCdel vs GCins/GCins; ^u^p < 0.0001 ACins/GCdel vs GGdel/ACins; ^w^p = 0.031 GCins/GCins vs GGdel/ACins;

**Men with abnormal sperm parameters**: ^v^p = 0.0313 GCdel/GCdel vs GCdel/GCins vs GCins/GCins vs (Kruskal-Wallis test);

**Asthenozoospermic men** **vs** **Asthenozoospermic men:** ^x^p = 0.0004 ACdel/ACdel vs GCins/GCins; ^y^p = 0.002 ACdel/ACdel vs GGdel/ACins; ^z^p = 0.003 ACins/GCdel vs GCins/GCins; ^aa^p = 0.053 ACins/GCdel vs GGdel/ACins;

**Teratozoospermic men vs Teratozoospermic men:** ^ab^p = 0.050 ACdel/ACdel vs ACdel/AGdel; ^ac^p = 0.012 ACdel/ACdel vs ACdel/GGdel; ^ad^p = 0.0004 ACdel/ACdel vs GCdel/GCins; ^ae^p < 0.0001 ACdel/ACdel vs GCins/GCins; ^af^p < 0.0001 ACdel/ACdel vs GGdel/ACins; ^ag^p = 0.011 ACdel/ACdel vs GTins/ACins; ^ah^p = 0.030 ACdel/AGdel vs GCdel/GCins; ^ai^p = 0.001 ACdel/AGdel vs GCins/GCins; ^aj^p = 0.010 ACdel/AGdel vs GGdel/ACins; ^ak^p = 0.022 ACdel/GCdel vs GCdel/GCins; ^al^p = 0.010 ACdel/GCdel vs GGdel/ACins; ^am^p = 0.006 ACdel/GGdel vs GCdel/GCins; ^an^p = 0.004 ACdel/GGdel vs GCins/GCins; ^ao^p = 0.034 ACdel/GGdel vs GGdel/ACins; ^ap^p = 0.003 ACins/GCdel vs GCdel/GCins; ^aq^p < 0.0001 ACins/GCdel vs GCins/GCins; ^ar^p = 0.0004 ACins/GCdel vs GGdel/ACins; ^as^p = 0.013 GCdel/GCins vs GCins/GCins; ^at^p = 0.026 GCins/GCins vs GGdel/ACins; ^au^p = 0.041 GCins/GCins vs GTins/ACins
